# Morphology Engineering of SnS_2_ Nanostructures to Stimulate PICT Resonance for Ultra‐Sensitive SERS Sensors

**DOI:** 10.1002/EXP.70016

**Published:** 2025-02-24

**Authors:** Yusi Peng, Weida Zhang, Meimei Xu, Shuai Zhao, Lili Yang, Dan Li, Masaki Tanemura, Zhengren Huang, Yong Yang

**Affiliations:** ^1^ State Key Laboratory of High‐Performance Ceramics and Superfine Microstructures Shanghai Institute of Ceramics Chinese Academy of Sciences Shanghai People's Republic of China; ^2^ University of Chinese Academy of Sciences Beijing People's Republic of China; ^3^ Center of Materials Science and Optoelectronics Engineering University of Chinese Academy of Sciences Beijing China; ^4^ College of Integrated Circuit Science and Engineering Nanjing University of Posts and Telecommunications Nanjing China; ^5^ Department of Frontier Materials Graduate School of Engineering Nagoya Institute of Technology Nagoya Japan

**Keywords:** morphology engineering, PICT resonance, SnS_2_ nanostructures, ultra‐high SERS sensitivity

## Abstract

Recent advances indicate the surface‐enhanced Raman scattering (SERS) sensitivity of semiconductors is generally lower than that of noble metal substrates, and developing ultra‐sensitive semiconductor SERS substrates is an urgent task. Here, SnS_2_ with better SERS performance is screened out from sulfides and selenides by density functional theory (DFT) calculations. Through adjusting the concentration of reactants to control the growth driving force without any surfactants or templates, SnS_2_ nanostrctures of stacked nanosheets (SNSs), microspheres (MSs) and microflowers (MFs) are developed, which all exhibit ultra‐low limit of detections (LODs) of 10^−12^, 10^−13^, and 10^−11^ M, respectively. To the best of our knowledge, the SERS sensitivity of these three kinds of SnS_2_ nanostrctures are superior to most of the reported pure semiconductors and even can be parallel to the noble metals with a “hot spot” effect. This extraordinary SERS enhancement of SnS_2_ nanostrctures is originated from the dominated contribution of photo‐induced charge transfer (PICT) resonance with different wavelength excitation lasers. Benefitting to the excellent SERS enhanced uniformity, generality, stability, ultra‐high sensitivity of SnS_2_ nanostrctures, and the advantages that the PICT resonance enhancement excited for different probe molecules is not limited by its morphology, it is expected to provide a class of potential commercial SERS‐active materials for the practical application of semiconductor‐based SERS technology.

## Graphical abstract

The developed SnS_2_ nanostrctures with three morphology of stacked nanosheets, microspheresand microflowers exhibit the excellent SERS enhanced uniformity, generality, stability and ultra‐high sensitivity, which are expected to provide a class of potential commercial SERS‐active materials for the practical application of semiconductor‐based SERS technology.

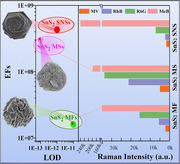

## 1 Introduction

As a trace spectroscopic detection technology, surface‐enhanced Raman scattering (SERS) is intensively applied in surface science, electrochemical analysis, environmental monitoring, cultural relic analysis and bio‐sensing and other fields due to its advantages of vibration fingerprint characteristics, high detection sensitivity, high accuracy, and non‐destructiveness. Undoubtedly, the enhanced substrate material with SERS activity is the key objective to further promote the development of SERS technology in addition to the SERS intrinsic enhancement mechanism. As the synchronous rapid development of nanotechnology and materials science, SERS materials have extended from Group 11 noble metals to semiconductor nanostructures [1, 2, 3]. The current research has reported a large number of semiconductor‐based SERS substrates, such as such as metal oxides, transition metal sulfides, metal nitrides, and metal carbides, silver halides [2, 3, 4, 5, 6]. Compared with noble metal SERS substrates dominated by electromagnetic enhancement (EM), semiconductor SERS substrates originating from chemical mechanism (CM) exhibit better spectral stability and biocompatibility [7], presenting great application potential. However, the SERS sensitivity of semiconductor substrates is generally lower than that of noble metal substrates, facing formidable challenges in some practical detection applications. Therefore, it is crucial to develop ultra‐sensitive semiconductor SERS substrates.

Currently, some commonly effective strategies have been developed to optimize the SERS performance of semiconductor substrates including introducing defect engineering, amorphization, alloy engineering, and morphology regulation [8]. Among them, the beneficial effect of defect engineering and amorphization strategies is mainly improving the photo‐induced charge transfer (PICT) efficiency to enhance the SERS performance contributed by CM through adjusting the intrinsic chemical properties such as the energy band structures of substrate materials [9, 10]. The alloy engineering strategy can be applied to adjust the energy levels of semiconductor materials over a wide range, so as to generate the coupling effect between the energy levels of a semiconductor‐molecule system and the laser wavelength, thus achieving a strong PICT resonance [11]. The morphology engineering can not only develop the physical adsorption capacity of substrates to molecules through adjusting the surface physical properties such as the specific surface area of substrate materials, but also improve the scattering efficiency of irradiation laser by constructing bowl or hole structures, and conduce to achieve the coupling enhancement of the SPR effect through assembling complex nanostructure with regular morphology [12, 13, 14]. The differences in SERS sensitivity of highly uniform Cu_2_O crystallites with three kinds of morphologies including cube, rhombic dodecahedron, and octahedron are mainly originated from the differences in the interfacial charge transfer of different morphologies [5]. The SERS sensitivity of Nb_2_O_5_ nanoparticles can be optimized by an order of magnitude through regulating their morphology into ultrathin nanosheets [15]. The γ‐Mo_2_N porous nanoribbons and δ‐MoN porous nanopillars with different crystal types synthesized by Xi's research group both presented strong SPR effect, and the “hot spot” effect in the high‐density nanopores can further enhance the Raman signal of molecules significantly [6, 13, 16]. Additionally, this group assembled the ultrathin and oxygen‐vacancy‐rich W_18_O_49_ nanowires with a thickness of about 1.5 nm into a highly ordered mesocrystal morphology with a size of about 100 nm. Benefiting from its mesocrystal structure, the local surface plasmon resonance (LSPR) enhancement effect and the efficiency of interfacial charge transfer between substrates and molecules are both conspicuously developed [17]. Much research achievements demonstrated that morphology regulation is an effective method to further optimize the SERS sensitivity of semiconductor substrates. However, usually, every coin has two sides. The repeatability and stability of SERS enhancement for ultra‐sensitive semiconductor substrates after well‐designed morphology optimization usually face challenges, and the great influence on its SERS performance will be caused by some slight changes in the morphology limited by the complex preparation process. Therefore, it is of great significance for the practical application of SERS technology to explore ultra‐sensitive semiconductor SERS substrates whose SERS performance can be optimized by morphology regulation but are not limited to slight changes in its morphology. Furthermore, another major advantage of semiconductor substrates is the material category and morphology are abundant and susceptible to regulation compared with noble metal substrates, which can promote the semiconductor‐based SERS technology toward a greater height.

Transition metal dichalcogenides and selenides (TMDCs), as typical two‐dimensional (2D) layered semiconductor materials, have shown great application potential in the SERS detection field. As far as research reported, various 2D sulfides and selenides with SERS activity such as MoS_2_, ReS_2_, WS_2_, WSe_2_, NbSe_2_, NbS_2_, SnS_2_, NbTe_2_ and ZnSe have been developed [3, 4, 18, 19]. Among them, Peng first developed an ultra‐sensitive SERS substrate SnS_2_ microspheres with an ultra‐low limit of detection (LOD) of 10^−13^ M for methylene blue (MeB) molecules [20]. Kitadai reported a kind of SnS_2_ nanostructure with exciton‐coupled SERS enhancement, which could also achieve a low LOD of 10^−13^ M for Rhodamine 6G (Rh 6G) molecules [21]. In order to further improve the SERS sensitivity of SnS_2_ nanostructures, researchers currently decorated Ag/Au nanoparticles on SnS_2_ nanosheets or quantum dots to achieve the rapid detection of dyes, heavy metal Hg (II) ions in the water environments, and selective quantification of methimazole in serum and meat samples [22]. The above research shows that the abundant electronic density of states (DOS) near the Fermi level of SnS_2_ will facilitate to development of the efficiency of charge transfer between molecules and substrates, and their atomically thin flat surface and high chemical stability will be beneficial to obtain the repeatable and stable Raman spectra in the practical detecting applications. Moreover, most of the interlayer interactions of SnS_2_ are dominated by the weak van der Waals force from long‐range interactions, which facilitates regulating their thickness and morphology structure. Above mentioned advantages endow SnS_2_ becoming the perfectly appropriate candidates to explore the influence of morphology structure on the SERS performance of semiconductor substrates and further develop the CM‐dominated ultra‐sensitive SERS substrates.

In this work, SnS_2_ crystal with better SERS performance was first screened out from 2D sulfides and selenides‐based density functional theory (DFT) calculations. Then, SnS_2_ nanostrctures with three kinds of morphologies were successfully synthesized as SERS substrates by adjusting the concentration of reactants to control the growth driving force without any surfactants or templates. The developed SnS_2_ stacked nanosheets (SNSs), SnS_2_ microspheres (MSs) and SnS_2_ micro‐flowers (MFs) exhibit ultra‐high SERS sensitivity with extremely low LODs of 10^−12^, 10^−13^, and 10^−11^ M for MeB molecules under the excitation laser of 785 nm, which exceeds the SERS sensitivity of most reported pure semiconductor substrates and even can parallel to the noble metal substrates with “hot spot” effect. Based on the analysis of energy band structures, the SERS enhancement mechanism of SnS_2_ nanostructure with three kinds of morphologies to different molecules is originated from the dominated contribution of PICT resonance with different wavelength excitation lasers. Moreover, these three kinds of SnS_2_ nanostrctures exhibit excellent uniformity, generality, and stability of SERS enhancement, which shows broad prospect in the practical application of SERS technology.

## 2 Materials and Methods

### 2.1 Screening out 2D Sulfides and Selenides With Better SERS Performance Based on DFT Calculations

The first‐principles calculations based on DFT [23] are employed to predict and compare the SERS activity of eight types of 2D sulfides and selenides, thus screening out the substrate materials with better SERS performance. First, we constructed the MeB molecule model and their complex adsorption models with eight kinds of 2D sulfides and selenides including MeB‐SnS_2_, MeB‐SnSe_2_, MeB‐ReS_2_, MeB‐ReSe_2_, MeB‐TiS_2_, MeB‐TiSe_2_, MeB‐VS_2_, MeB‐VSe_2_, MeB‐WS_2_, MeB‐WSe_2_, MeB‐MoS_2_, MeB‐MoSe_2_, MeB‐NbS_2_, MeB‐NbSe_2_, MeB‐ZnS, and MeB‐ZnSe. Then, the Gauss09 program was adopted to complete the ground state geometry optimization, static Raman spectra, highest occupied molecular orbital (HOMO) and lowest unoccupied molecular orbital (LUMO) energy level distribution of the above calculation models. Here, Becke's three‐parameter mixed exchange function and the Lee, Yang, Parr (B3LYP) exchange function combined with basis sets were applied for all calculations. To ensure keeping all calculation structures in a stable state with the lowest energy, the probe molecule and adsorption models were optimized without virtual frequencies. The 6–311+G (d, p) group with polarization function and diffusion function was selected for the C, H, O, N, S, and Se atoms. The transition metals Sn, Re, Ti, V, W, Mo, Nb, and Zn atoms were described by the Lanl2dz basic group.

### 2.2 Synthesis of SnS_2_, VS_4_, VS_2_, and MoS_2_ Nanostructures

#### 2.2.1 SnS_2_ Nanostructures

The SnS_2_ nanostructures with three kinds of morphologies were synthesized by a simple one‐step hydrothermal reaction without any surfactants or templates. The growth driving force of SnS_2_ nanosheets was controlled by adjusting the concentration of reactants, thereby synthesizing the SnS_2_ nanostructures with three kinds of morphologies including hexagonal stacked nanosheet, microsphere, and micro‐flower. First, thioacetamide (TTA, 0.8, 1.6, and 2.4 g) were dissolved into deionized water (55 mL), respectively, and magnetically stirred at 60°C to obtain the mixed transparent solutions. Then, Na_2_SnO_3_·3H_2_O powder (0.4, 0.8, and 1.2 g) was dissolved in the above TTA transparent solutions, respectively, and magnetically stirred to obtain the uniform precursor solution. Next, the above precursor solutions were transferred into the PPL hydrothermal reactor (100 mL), and conducted the hydrothermal reaction at 180°C for 24 h. Finally, the yellow, brown, and dark brown precipitates were obtained, respectively, and washed at least three times with deionized water as well as freeze‐dried to obtain sample powder including yellow SnS_2_ SNSs, brown SnS_2_ MSs, and dark brown SnS_2_ MFs, respectively.

#### 2.2.2 VS_4_, VS_2_, and MoS_2_ Nanostructures

Na_3_VO_4_ powder (0.736, 1.472, and 2.208 g) and TTA (1.5, 3.0, and 4.5 g) were dissolved into deionized water (60 mL), respectively, and conduct the hydrothermal reaction at 160°C for 20 h to obtain the VS_4_ nanostructure powder. Similarly, NH_4_VO_3_ powder (0.7, 1.4, and 2.1 g) and TTA (2.4, 4.8, and 7.2 g) were dissolved into the mixture solution of deionized water (45 mL) and ammonia liquor (NH_3_·H_2_O, 9 mL), respectively, and conduct the hydrothermal reaction at 160°C for 20 h to obtain the VS_2_ nanostructure powder. MoS_2_ nanostructure powder was obtained by dissolving ammonium molybdate tetrahydrate ((NH_4_)_6_Mo_7_O_24_·4H_2_O) powder (1.6, 3.2, and 4.8 g) and thiourea (CH_4_N_2_S, 2.0, 4.0, and 6.0 g) in deionized water (60 mL) as precursors to conduct hydrothermal reaction at 180°C for 12 h.

### 2.3 Characterizations and SERS Measurements

Here, the X‐ray diffraction (XRD) spectra of SnS_2_ nanostructure with three kinds of morphologies were measured by using the Rigaku D/MAX‐2200 PC XRD system (parameters: Cu Kα radiation, *λ* = 1.54 Å, 40 mA, and 40 kV). The FEI Magellan 400 field emission scanning electron microscopy (FESEM) was used to provide the micro‐morphology of the SnS_2_ nanostructure with three kinds of morphologies. The transmission electron microscopy (TEM), high‐resolution TEM (HRTEM), energy‐dispersive X‐ray spectroscopy (EDS) and selected area electron diffraction (SAED) images of SnS_2_ nanostructure with three kinds of morphologies were obtained by the JEM‐2100F field emission source transmission electron microscope (200 kV). The thickness of SnS_2_ hexagonal SNSs was observed by atomic force microscopy (AFM) images measured by the Veeco DI Nanoscope Multi Mode V system. The valence state information of SnS_2_ nanostructure with three kinds of morphologies was analyzed by X‐ray photoelectron spectroscopy (XPS) measured by Thermo Fisher Scientific ESCAlab250.

In order to explore the SERS performance of SnS_2_ nanostructure with three kinds of morphologies to the MeB molecules with different concentrations of 10^−6^–10^−13^ M were adopted for SERS detection. As for each Raman test, the synthesized SnS_2_ sample powders (0.01 g) with three kinds of morphologies were immersed in MeB molecule aqueous solution (30 mL) and treated with ultrasound for 2 h. A dose of mixture solution with a volume (5 µL) in the bottom of the tube was collected by centrifugation and dropped on the surface of the glass substrate as well as dried at room temperature. All Raman spectra were detected by a Renishaw in Via Reflex Raman spectrometer, and followed the same detection conditions: the irradiation power of 0.5 mW for 532 nm laser, 0.17 mW for 633 nm laser, and 0.15 mW for 785 nm laser, the accumulation time of 20 s, and the laser beam of 50× microscope objective. At least three different points were tested on each molecule‐substrate complex, and the Raman peak at 1620 cm^−1^ was selected to calculate the SERS enhancement factor (EF) value as well as to analyze the relationship trend between Raman intensity and MeB concentration.

## 3 Results and Discussion

### 3.1 Screening of 2D Sulfides and Selenides With Better SERS Activity Based on DFT Calculations

Motivated by the rapid development of 2D semiconductor materials, the emerging TMDCs provide some competitive candidates for the ultra‐sensitive SERS substrates. However, the SERS detection sensitivity of different TMDCs varies greatly from 10^−6^ to 10^−14^ M [20]. Therefore, screening out substrate materials with better SERS performance from TMDCs in advance based on DFT calculations is the more efficient approach to achieving the ultrasensitive semiconductor‐based SERS detection platform. Here, eight kinds of common and successfully synthesized TMDCs were selected, which are SnS_2_, SnSe_2_, ReS_2_, ReSe_2_, TiS_2_, TiSe_2_, VS_2_, VSe_2_ with space group of $P\bar{3}m1$, and WS_2_, WSe_2_, MoS_2_, MoSe_2_, NbS_2_, NbSe_2_ with space group of P6_3_/mmc, as well as ZnS, ZnSe with space group of P6_3_mc. The traditional molecule MeB with one strongest characteristic Raman peak was selected to evaluate the SERS performance of the above‐mentioned TMDCs substrates.

**FIGURE 1:**
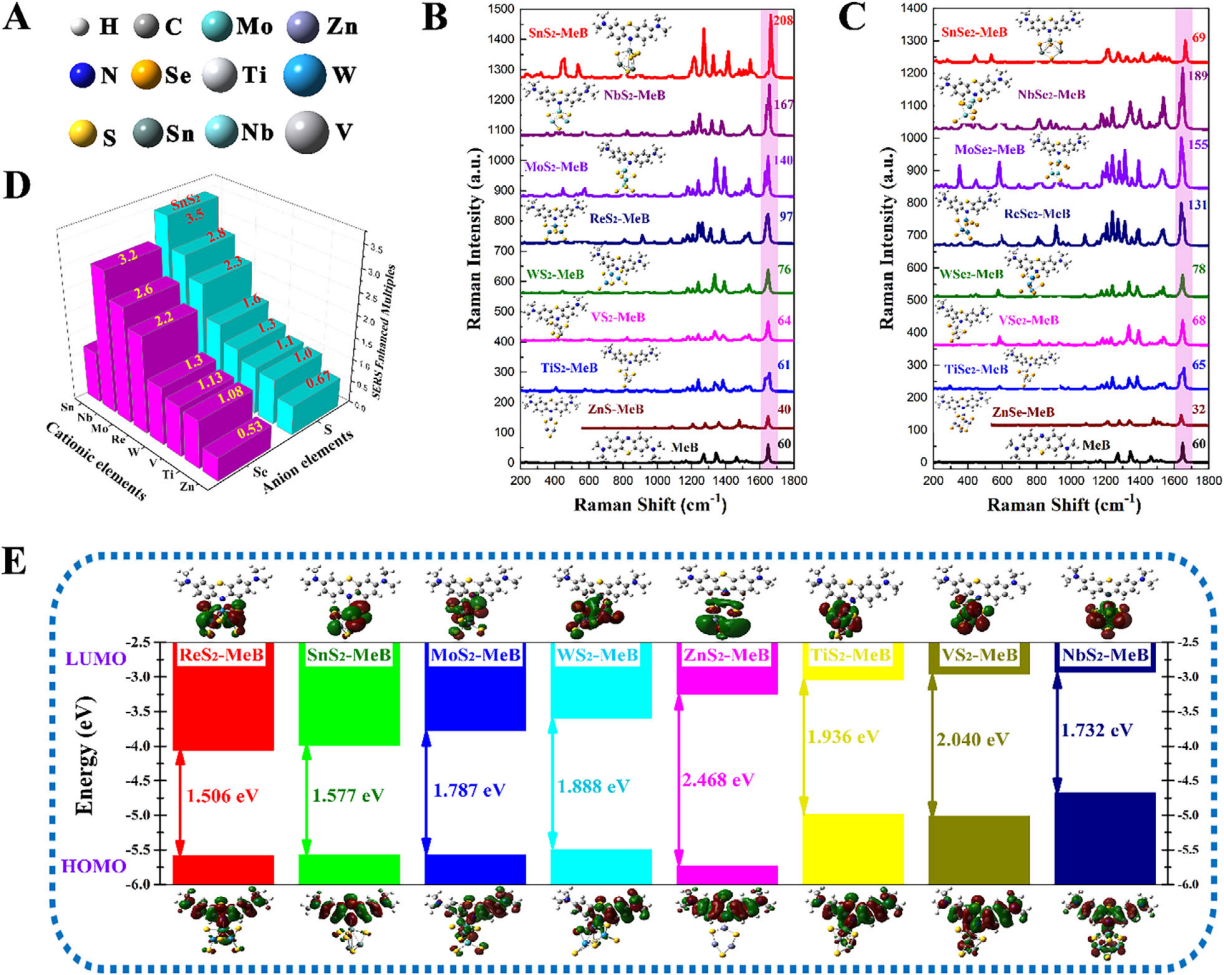
Screening the materials with more excellent SERS activity among eight kinds of common sulfides and selenides based on DFT calculation of static Raman spectra. (A) Schematic diagram of different colored balls representing different elements. (B) Static Raman spectra of MeB molecules adsorbed on eight kinds of sulfides (SnS_2_, NbS_2_, MoS_2_, ReS_2_, WS_2_, VS_2_, TiS_2_, and ZnS_2_). (C) Static Raman spectra of MeB molecules adsorbed on eight kinds of selenides (SnSe_2_, NbSe_2_, MoSe_2_, ReSe_2_, WSe_2_, VSe_2_, TiSe_2_, and ZnSe_2_). (D) The comparison of Raman enhanced multiples for MeB‐sulfides and MeB‐selenides. (E) The energy level distributions and HOMO/LUMO illustrations of MeB‐sulfides.

It is well‐known that the Raman enhancements of 2D semiconductor‐based SERS substrates mainly originated from the CM mechanism. Therefore, the static Raman spectra and HOMO/LUMO energy level distribution of the substrate‐molecule complex were calculated to characterize the contribution of CM to SERS activity of TMDCs substrates and determine its optimal resonance excitation wavelength. First, we constructed the MeB molecule model and their complex adsorption models with eight kinds of 2D TMDCs including MeB‐SnS_2_, MeB‐SnSe_2_, MeB‐ReS_2_, MeB‐ReSe_2_, MeB‐TiS_2_, MeB‐TiSe_2_, MeB‐VS_2_, MeB‐VSe_2_, MeB‐WS_2_, MeB‐WSe_2_, MeB‐MoS_2_, MeB‐MoSe_2_, MeB‐NbS_2_, MeB‐NbSe_2_, MeB‐ZnS, MeB‐ZnSe. The balls with different colors represent different kinds of atoms (Figure 1A). Here, MeB molecules are bonded to SnS_2_, SnSe_2_, ReS_2_, ReSe_2_, TiS_2_, TiSe_2_, VS_2_, VSe_2_, WS_2_, WSe_2_, MoS_2_, MoSe_2_, NbS_2_, NbSe_2_, ZnS, and ZnSe clusters via Sn‐N, Re‐N, Ti‐N, V‐N, W‐N, Mo‐N, Nb‐N, and Zn‐N bonds, respectively. Their calculated static Raman spectra are shown in Figure 1B,C. In addition to ZnS and ZnSe substrates, the Raman signals of MeB molecules in the calculated static Raman spectra are all enhanced to varying degrees after adsorbing to other seven kinds of TMDCs (Figure 1D). According to the Raman enhancement multiples of MeB molecules on eight kinds of TMDCs, it can be clearly seen that SnS_2_ and NbSe_2_ exhibit stronger SERS enhancement to MeB molecules. Among them, the SERS performance of NbSe_2_ has been reported, and the detection of limit for R6G molecules can be as low as 5 × 10^−16^ M after the nanosheets reduce to six layers [3]. While SnS_2_ exhibit even better SERS enhancement for MeB molecules than the NbSe_2_ cluster in the calculated static Raman spectra, indicating that the SnS_2_ nanostructure is expected to SERS substrates with ultra‐high detection sensitivity.

In order to further analyze the SERS‐enhancing mechanism of MeB molecules on eight kinds of TMDCs substrates and determine their optimal excitation wavelengths originating from the charge transfer resonance, the HOMO/LUMO energy level distribution was calculated. When the MeB molecule was adsorbed on the TMDCs substrates, the metal atom‐N chemical bonds can be formed as charge transfer channels to promote the charge transfer, thereby resulting in a rearrangement of electron clouds of MeB molecules and 2D sulfide and selenide clusters. As shown in Figure 1E and Figure S1, the accumulated regions of electron density on the HOMO energy levels of the MeB‐substrate complexes are mainly localized on the MeB molecules or the bonding orbitals of N atoms in MeB molecules and metal atoms in substrates, while the depleted regions of electron density on the LUMO energy levels mainly span the TMDCs clusters, indicating that the electrons are susceptible to transfer from the MeB molecules to the conduction band of TMDCs substrates. Compared with the HOMO‐LUMO energy gap of 3.48 eV for MeB molecule, the rearrangement of electrons in the MeB‐substrate complexes endow their band gap with varying degrees of decrease. Their optimal excitation wavelengths dominated by the PICT process are all adjusted from the ultraviolet region (<380 nm) to the visible region (380–780 nm). Therefore, the charge‐transfer efficiency of these eight kinds of TMDCs substrates can be significantly improved under the excitation of a specific wavelength of incident laser, even can generate PICT resonance. Among these eight kinds of TMDCs substrates, MeB‐ZnS and MeB‐ZnSe complexes can be excited more efficiency by the excitation laser of 532 nm, while MeB‐MoS_2_ and MeB‐MoSe_2_, MeB‐WS_2_ and MeB‐WSe_2_, MeB‐TiS_2_ and MeB‐TiSe_2_, MeB‐VS_2_ and MeB‐VSe_2_ complexes can generate more obvious Raman enhancement with the excitation laser of 633 nm. Moreover, MeB‐TiS_2_ and MeB‐VSe_2_ complexes may even produce PICT resonance under the excitation laser of 633 nm. MeB‐SnS_2_ and MeB‐SnSe_2_, MeB‐ReS_2_ and MeB‐ReSe_2_, and MeB‐NbS_2_ and MeB‐NbSe_2_ complexes can be more effectively excited by the excitation laser of 785 nm to generate enhanced Raman signals. Especially for the MeB‐SnS_2_ complex, its HOMO‐LUMO energy gap of 1.577 eV is much closer to the excitation energy of 785 nm laser, indicating that the MeB‐SnS_2_ complex can generate an efficient PICT resonance under the excitation laser of 785 nm, thus showing the excellent SERS enhancement. In summary, SnS_2_ substrate with better SERS performance was successfully screened out through DFT calculations, and determined that its optimal excitation wavelength originated from the PICT resonance for MeB molecules is 785 nm, which provides an effective theoretical guidance for subsequent SERS performance optimization of SnS_2_ materials.

### 3.2 Characterization of SnS_2_ Nanostructure With Three Kinds of Morphologies

**FIGURE 2:**
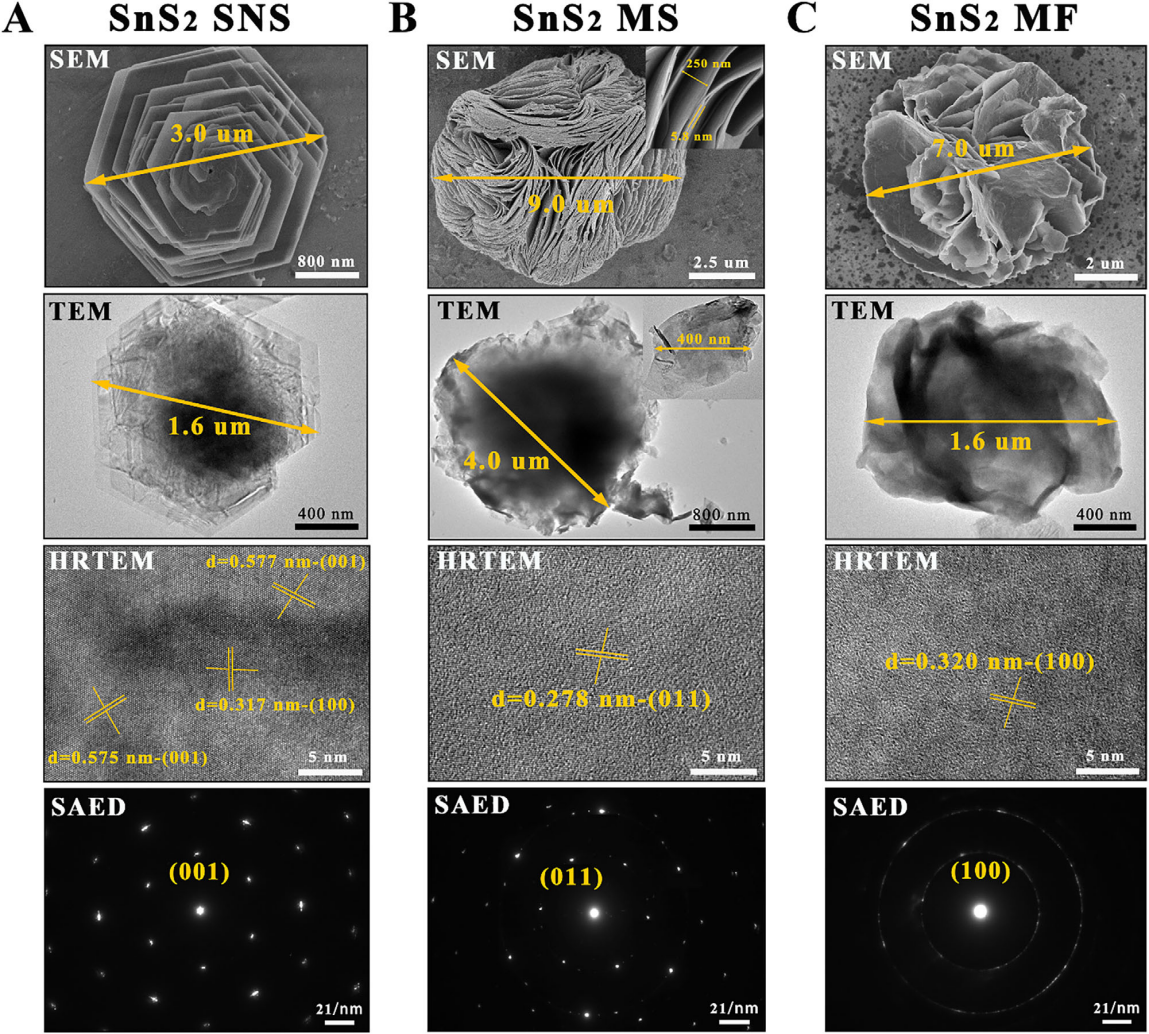
Morphological characterization of SnS_2_ nanostructure with three kinds of morphologies. (A–C) SEM images, TEM images, HRTEM images, and their corresponding SAED images of SnS_2_ SNSs (A), SnS_2_ MSs (B) and SnS_2_ MFs (C).

Here, SnS_2_ nanostructure with three kinds of morphologies was successfully synthesized by a simple one‐step hydrothermal reaction. The SEM, TEM, HRTEM, and SAED images were applied to comprehensively investigate the morphology structure of samples, which are shown in Figure 2A–C. The morphologies of these three kinds of synthesized SnS_2_ powder with colors of yellow, brown, and dark brown are the regular hexagonal helical stacked layered structure, the special spherical structure formed by the curling stacked nanosheets and the flower‐like structure assembled by nanosheets, which are named stacked nanosheets (SNSs), microspheres (MSs) and microflowers (MFs), respectively. The transverse dimensions of these three kinds of SnS_2_ nanostructures all can reach the micrometer scale. AFM image shows (Figure S2) that the thickness of SnS_2_ SNS is 1.5–2.0 µm. SnS_2_ MS is an unreported novel morphology with the special nano‐“canyon” hierarchical nanostructure exists on the surface of microspheres [20]. The clear lattice diffraction fringes with the inter‐planar spacing of 0.576 nm on SnS_2_ SNSs, 0.278 nm on SnS_2_ MS and 0.32 nm on SnS_2_ MF can be easily discerned by their HRTEM images, which correspond to the (001) plane, (011) plane, and (100) plane of the hexagonal SnS_2_ crystal (Figure S3), respectively. The SAED images of SnS_2_ SNS and SnS_2_ MS confirm the excellent crystallinity and the hexagonal symmetry structure, while the SAED image of SnS_2_ MF tends to form the sharp polycrystalline diffraction rings due to the growing of nanosheets along different orientations and its relatively poor crystallinity. Additionally, there are no other impurity signals except the S and Sn signals in their EDS spectra shown in Figure S4, demonstrating the extremely high purity of SnS_2_ SNSs, SnS_2_ MSs, and SnS_2_ MFs. Moreover, the Sn/S atomic ratios of these three kinds of SnS_2_ nanostructures are all approximately 1:2, which corresponds to the phase structure of SnS_2_.

**FIGURE 3:**
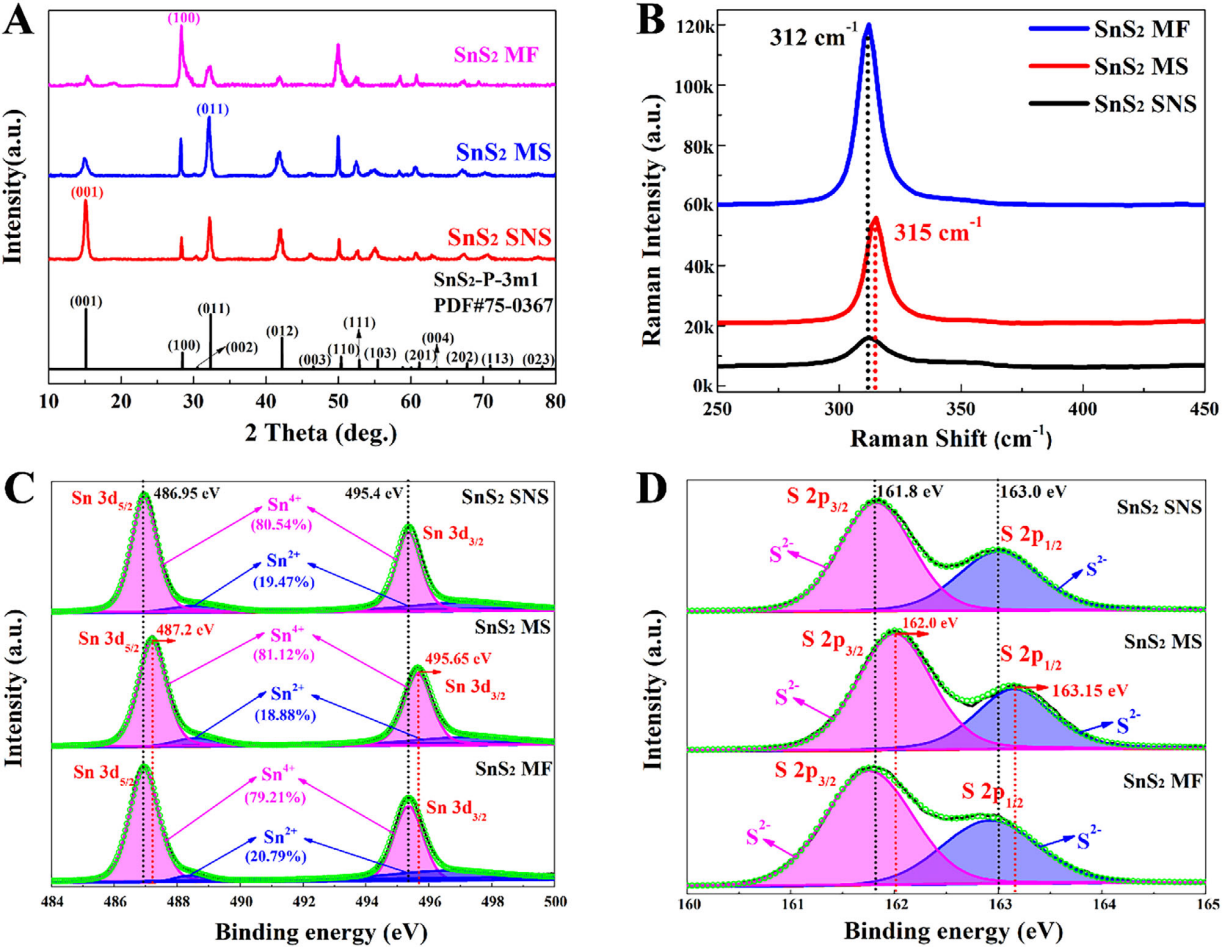
Phase structural characterization of SnS_2_ nanostructure with three kinds of morphologies. (A,B) XRD spectrum (A) and Raman spectra (B) of SnS_2_ SNSs, SnS_2_ MSs, and SnS_2_ MFs. (C,D) Sn3d (C) and S2p (D) XPS spectrum of SnS_2_ SNSs, SnS_2_ MSs and SnS_2_ MFs.

X‐ray diffraction (XRD) and X‐ray photoelectron spectroscopy (XPS) were further used to obtain more detailed information about the phase structure and superficial chemical state of SnS_2_ SNSs, SnS_2_ MSs, and SnS_2_ MFs. As shown in Figure 3A, these three kinds of SnS_2_ nanostructures are crystallized SnS_2_ (PDF#75‐0367) phases with lattice constants corresponding to the hexagonal crystal with the space group of $P\bar{3}m1$ (*a* = 3.65 Å, *b* = 3.65 Å, *c* = 5.90 Å). Although SnS_2_ SNSs, SnS_2_ MSs, and SnS_2_ MFs collectively belong to the same set of XRD peaks, their strongest XRD peaks are located at different 2*θ*s of 15.13°, 32.22° and 28.34°, respectively, which correspond to (001), (011) and (100) planes. It is indicated that SnS_2_ nanostructures with three kinds of morphologies exhibit different exposed crystal planes of (001), (011) and (100), which are consistent with the analysis results of HRTEM images. Furthermore, as previously discussed in HRTEM images, the XRD peaks of SnS_2_ SNSs and SnS_2_ MSs also both reveal an extremely excellent crystallinity, but SnS_2_ MFs are not. Raman spectra in Figure 3B show that these three kinds of SnS_2_ nanostructures all exhibit a distinguished Raman peak assigned to the vertical plane vibration mode (*A*_1g_) of Sn‐S bonds. Among them, this Raman peak of SnS_2_ SNSs and SnS_2_ MFs is located at 312 cm^−1^, while that of SnS_2_ MSs has an obvious red‐shift to 315 cm^−1^, which demonstrates the increase in interlayer distance of SnS_2_ nanosheets for microsphere morphology [24]. According to the XPS spectra of SnS_2_ SNSs, SnS_2_ MSs, and SnS_2_ MFs (Figure 3C,D), the Sn and S elements can be clearly identified in SnS_2_ nanostructures. in addition to the double peaks at 487.2 and 495.7 eV arising from Sn^4+^, there were two unambiguous doublet peaks located at 488.7 eV (Sn3d_5/2_) and 497.5 eV (Sn*3*d_3/2_), which belonged to Sn^2+^. The enlarged characteristic peaks in the S2p region located at 162.0 and 163.2 eV representing S2p_3/2_ and S2p_1/2_ were both attributed to S^2+^. The content ratio of Sn^4+^ and Sn^2+^ reflected by Sn3d XPS spectra is about 4:1, which indicates the existence of sulfur vacancies (V_S_) in these three kinds of SnS_2_ nanostructures. Among them, the content of V_S_ in SnS_2_ MFs is relatively larger, and a large number of V_S_ defects will destroy the crystallinity of materials, resulting in the relatively weak crystallinity of SnS_2_ MFs, which corresponds to the polycrystalline diffraction rings in the SAED image of SnS_2_ MFs. Theoretically, the weaker crystallinity and the more V_S_ defects in SnS_2_ MFs will affect the HOMO and LUMO energy levels, which can directly affect the PICT resonance strength between SnS_2_ nanostructures and probe molecules, thus showing different SERS enhancement effects. Furthermore, it is noteworthy that the Sn3d and S2p XPS peaks of SnS_2_ MSs exhibit an obvious shift to the higher binding energy in relation to SnS_2_ SNSs and SnS_2_ MFs, demonstrating the changed electron density difference of SnS_2_ MSs affected by the existence of lattice strain [25].

### 3.3 Formation Mechanism of SnS_2_ Nanostructure With Three Kinds of Morphologies

In this work, SnS_2_ nanostructures with three kinds of morphologies including SnS_2_ SNSs, SnS_2_ MSs, and SnS_2_ MFs were synthesized by adjusting the concentration of reactants to control the growth driving force of SnS_2_ nanosheets without any surfactants or templates. In order to further investigate the growth mechanism of these three kinds of SnS_2_ nanostructures with different morphologies, the hydrothermal reaction time of 2, 8, 16, and 24 h was adjusted to obtain their intermediate products. The corresponding SEM images and XRD spectrum of SnS_2_ SNSs, SnS_2_ MSs, and SnS_2_ MFs with the different hydrothermal reaction times are shown in Figures S5 and S6. In the growth process of SnS_2_ SNSs, SnS_2_ MSs, and SnS_2_ MFs, the concentration of reactants is the only one variable, which is controlled to be 1:2:3. Among them, the mass ratio of Na_2_SnO_3_·3H_2_O and TTA in every reaction is controlled to be 2:1. Because the growth rate of different crystal planes is mainly dominated by the off‐equilibrium driving force generated by the under‐cooling or super‐saturation of reactants. It is reasonable to think that the different concentrations of reactants are the growth‐driving forces of SnS_2_ nanostructures that grow into different morphologies along different crystal planes [26]. In the case of low concentrations of reactants, the growth process of SnS_2_ nanosheets will follow the spiral growth model due to the super‐cooled saturation. The growth step source that existed on the (001) crystal planes of SnS_2_ endows the nanosheets with the continuously spirally grown driving force and eventually grows into a spirally stacked nanosheets morphology.

There are mainly four processes involving nucleation, growth aggregation, self‐assembly and Ostwald maturation in the hydrothermal reaction process of growing SnS_2_ SNSs, SnS_2_ MSs, and SnS_2_ MFs. In the early stage of hydrothermal reactions, TTA (NH_2_CSNH_2_) is hydrolyzed to release H_2_S (S^2−^) involving the chemical reaction: CH_3_CSNH_2 _+ H_2_O = CH_3_CONH_2 _+ H_2_S, resulting in the weakly acidic initial aqueous medium environment. In this initial acidic medium with S^2−^ irons, the initial nucleation of SnS_2_ nanostructures is induced by the fact that the added Na_2_SnO_3_·3H_2_O provides the Sn source and occurs chemical reaction: Na_2_SnO_3 _+ 2H_2_S = SnS_2_↓ + 2NaOH + H_2_O. The generated SnS_2_ with lamellar morphology controlled by different crystal planes is attached to the SnS_2_ main nucleus, resulting in the growth aggregation of SnS_2_. The initial nucleation morphologies of these three kinds of SnS_2_ nanostructures have already been different after hydrothermal reaction 2 h (SEM images in Figure S5), and the corresponding XRD spectrum reveals a large amount of unreacted S elements with the space group of Fddd in the initial SnS_2_ samples (Figure S6). Then, the initial SnS_2_ nucleation grows along different orientations due to the different degrees of off‐equilibrium driving force generated by the different super‐saturations of reactants, thereby growing into different 3D SnS_2_ nanostructures constructed by the aggregation of nanosheets. SEM images after hydrothermal reaction of 8 h show that the morphologies of SnS_2_ SNSs, SnS_2_ MSs, and SnS_2_ MFs driven by different reactant saturations have begun to take shape. And the unreacted S elements in SnS_2_ samples have completely disappeared, while the complete characteristic diffraction peaks of SnS_2_ with space group of $P\bar{3}m1$ appears in the XRD spectrum after hydrothermal reaction of 8 h. Finally, SnS_2_ nanostructures with various sizes and morphologies are generated after the Ostwald ripening process with the further extension of the hydrothermal reaction time. SEM images after hydrothermal reaction of 16 h show the SnS_2_ SNSs with regular hexagonal nanosheets morphology, the SnS_2_ MSs with more geometric spherical morphology, and the SnS_2_ MFs with more perfect flower morphology. Further extending the hydrothermal reaction time to 24 h, the edges of SnS_2_ SNSs with more regular hexagonal morphology are sharpened significantly. SnS_2_ nanosheets in microspheres and microflowers grow further along the (011) and (100) directions, which endows SnS_2_ MSs and SnS_2_ MFs with larger sizes and more complete morphology. More specifically, the “canyon” morphology formed by the curling nanosheets on the surface of microspheres is more obvious, and the edges of nanosheets in microflowers generate significant sharpening. In conclusion, the regular SnS_2_ SNSs, SnS_2_ MSs, and SnS_2_ MFs are successfully synthesized by adjusting the growth driving force of nanosheets.

In the synthesis process of SnS_2_ SNSs, SnS_2_ MSs, and SnS_2_ MFs, the hydrothermal reaction time and temperature are also the two key regulatory factors in addition to the reactant concentration. Initiated from this view, some SnS_2_ nanostructures with three morphologies of hexagonal stacked nanosheets, microspheres and microflowers morphology were synthesized through regulating the hydrothermal reaction temperature to 150 and 240°C, as well as extending the hydrothermal reaction time to 36 h. As shown in SEM images and SERS enhanced Raman spectra of Figure S7, the morphology of SnS_2_ nanostructures synthesized at a hydrothermal reaction temperature of 150°C developed irregularly due to the restriction of reaction driving force, and the thickness of the nanosheets composed of these SnS_2_ nanostructures synthesized at hydrothermal reaction temperature of 240°C increased significantly and the nanosheets were more tightly stacked, resulting in the relative lower SERS sensitivity than that of SnS_2_ nanostructures synthesized at the optimal hydrothermal reaction temperature of 180°C. Further extending the hydrothermal reaction time to 36 h, the nanosheets of these synthesized SnS_2_ SNSs, SnS_2_ MSs, and SnS_2_ MFs significantly stacked more tightly due to the more adequate hydrothermal reaction, and their corresponding SERS sensitivity was significantly lower either. In conclusion, in addition to reactant concentration, the hydrothermal reaction temperature, and time are two key factors for the synthesis of SnS_2_ SNSs, SnS_2_ MSs, and SnS_2_ MFs while maintaining their excellent SERS performance and structural integrity. Additionally, six batches of SnS_2_ SNSs, SnS_2_ MSs, and SnS_2_ MFs were synthesized by hydrothermal reaction at the optimal reaction temperature of 180°C and reaction time of 24 h to explore the reproducibility of the synthesis process. SEM images (Figure S8) show the almost uniform morphology of stacked nanosheets, microspheres and microflowers in these six synthesis batches of SnS_2_ nanostructures. The Raman spectra of thirty random detection points on different synthesis batches of SnS_2_ nanostructures for 10^−8^ M MeB molecules exhibit excellent SERS enhanced uniformity and repeatability, indicating the good reproducibility of the synthesis process for SnS_2_ nanostructures in terms of excellent SERS performance and structural integrity.

Furthermore, another common TMDCs materials of vanadium sulfide and molybdenum sulfide were selected to explore the potential scalability of the synthesis process that regulates the morphology of products by controlling the concentration of reactants. Similar to the synthesis process of SnS_2_ nanostructures, the concentration of reactants is the only variable, which is controlled to be 1:2:3. SEM images (Figure S9) demonstrated the successful synthesis of VS_4_, VS_2_, and MoS_2_ nanostructures with the distinct morphologies. As for MoS_2_ nanostructures, with the increase of reactant concentration, MoS_2_ nanosheets agglomerate and form porous nanostructures. With respect to VS_4_ nanostructures, the thickness of VS_4_ nanosheets gradually increases with the increase of reactant concentration, and finally formed the agglomeration morphology of small particles. With regard to VS_2_ nanostructures, the VS_2_ nanosheets are stacked more tightly with the increase of reactant concentration, and the microflower morphology transfers into the microsphere morphology with the tightly stacked nanosheets. The above research indicated that the morphology of VS_4_, VS_2_, and MoS_2_ nanostructures is significantly affected by the change of reactant concentration. Unfortunately, VS_4_, VS_2_, and MoS_2_ nanostructures with three morphologies showed an unsatisfactory SERS enhancement effect (Figure S9), which may have originated from the lack of designable regulation for optimal hydrothermal reaction time as well as temperature and reactant concentration in the synthesis process. In conclusion, the obvious changes of morphology in VS_4_, VS_2_, and MoS_2_ nanostructures at different reactant concentrations demonstrated the potential scalability of this synthesis process that regulates the morphology of products through controlling the concentration of reactants.

### 3.4 SERS Performance for SnS_2_ Nanostructure With Three Kinds of Morphologies

**FIGURE 4:**
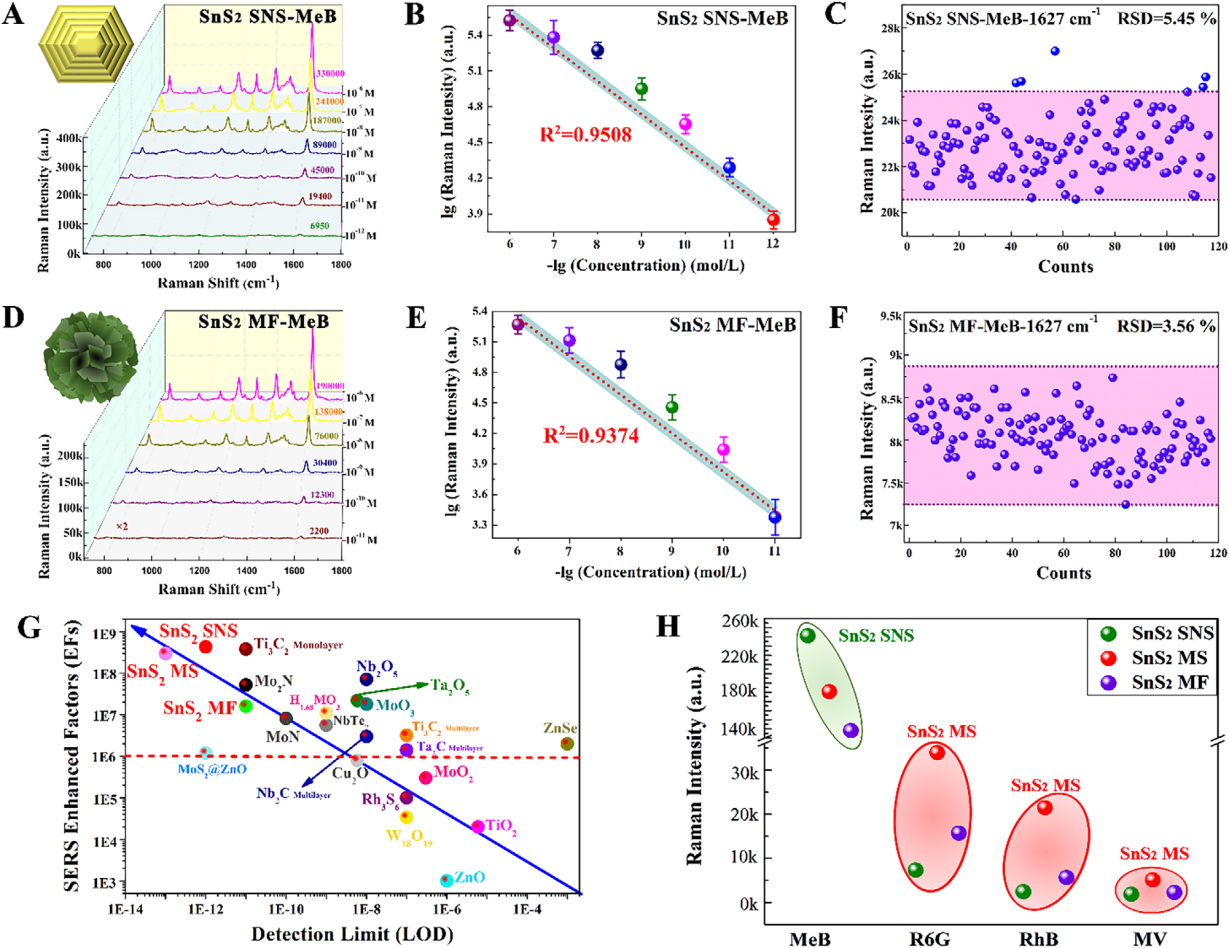
SERS performance of SnS_2_ nanostructure with three kinds of morphologies. (A,B) Raman spectra (A) of MeB with a concentration of 10^−6^–10^−12^ M on SnS_2_ SNSs and its relationship between Raman intensity and molecular concentration (B). (D,E) Raman spectra (D) of MeB with a concentration of 10^−6^–10^−11^ M on SnS_2_ MFs and its relationship between Raman intensity and molecular concentration (E). (C,F) The scatter plots of Raman intensity at 1627 cm^−1^ obtained on 118 test points from SnS_2_ SNSs (C) and SnS_2_ MFs (F) substrates. (G) Comparison of the EFs and LODs for SnS_2_ nanostructure with three kinds of morphologies in this work and other reported semiconductor‐based SERS substrates [10, 12, 15, 18, 27]. (H) Raman Intensity of 10^−7^ M MeB, MV, R6G and RhB on SnS_2_ SNSs, SnS_2_ MSs, and SnS_2_ MFs.

Herein, the traditional dye molecules of MeB, which exhibited obvious Raman characteristic peaks and could be excited by the visible laser, were selected to evaluate the SERS performance of above three kinds of SnS_2_ nanostructures with different morphology. The optimal excitation wavelengths of SnS_2_ SNSs, SnS_2_ MSs, and SnS_2_ MFs substrates on MeB molecules were determined for the first, and the Raman spectra are shown in Figure S10A–C. It can be clearly identified that the irradiation laser of 785 nm can better excite the MeB‐SnS_2_ nanostructure complexes to generate a more significant Raman signal relative to the irradiation lasers of 532 and 633 nm. Hence, the optimal excitation wavelength of SnS_2_ nanostructures with three kinds of morphologies for MeB molecules is determined to be 785 nm, which is consistent with the theoretical prediction results. Furthermore, it can also be seen in the Raman spectra with the excitation laser of 785 nm (Figure S10D) that the SERS enhancement effect of SnS_2_ nanostructures with three kinds of morphologies on the high concentration of MeB molecules (10^−6^ M) is follows: SnS_2_ SNSs > SnS_2_ MSs > SnS_2_ MFs. It is well acknowledged that ultra‐high detection sensitivity is crucial importance for expanding the application of semiconductor‐based SERS technology. Hence, the LOD of SnS_2_ SNSs, SnS_2_ MSs, and SnS_2_ MFs substrates on MeB molecules with the concentrations of 10^−6^–10^−13^ M were investigated, and their corresponding Raman spectra and the variation trend of Raman intensity with molecular concentration were shown in Figure 4A,D and referred to the previous reported work [20]. The LODs of SnS_2_ SNSs, SnS_2_ MSs, and SnS_2_ MFs substrates for MeB molecules under the excitation laser of 785 nm are as low as 10^−12^, 10^−13^, and 10^−11^ M, respectively, which all break through the new encountered detection bottleneck of 10^−10^ M for pure semiconductor SERS substrates, showing the outstanding SERS sensitivity. Moreover, it can be clearly seen from Figure 4B,E that the Raman intensity of SnS_2_ SNSs and SnS_2_ MFs on MeB molecules exhibits a linear relationship with the molecule concentration in the range of 10^−6^ to 10^−12^ M and 10^−6^ to 10^−11^ M with the correlation coefficients of 0.9508 and 0.9304, respectively. Unfortunately, their linear correlations are both relatively poor, which may be originated from the non‐uniform adsorption of MeB molecules on SnS_2_ nanostructures powder and the randomness of Raman test points. While the Raman intensity of MeB molecules on SnS_2_ MSs no longer decreases linearly with the decrease of molecular concentration of 10^−6^–10^−13^ M, which is mainly contributed to the molecular enrichment phenomenon caused by the capillary effect on the surface of microspheres [20]. The corresponding SERS EFs of SnS_2_ SNSs, SnS_2_ MSs, and SnS_2_ MFs on MeB molecules at Raman shifts of 1617 cm^−1^ is 4.3 × 10^8^, 1.6 × 10^7^, and 3.0 × 10^8^, respectively. As far as the currently reported literatures, the SERS performance of SnS_2_ SNSs, SnS_2_ MSs, and SnS_2_ MFs are more excellent than the most of pure semiconductor substrates (Figure 4G and Table S1), even can parallel to the noble metal substrates with “hot spot” effect. Among them, the EF calculation of SnS_2_ MSs takes into account a 40‐fold molecular physical enrichment [20].

Then, the uniformity, stability and generality of SERS enhancement were investigated to evaluate the usefulness of SnS_2_ SNSs, SnS_2_ MSs, SnS_2_ MFs SERS substrates. Raman signals of 118 detected points on the surface of MeB‐SnS_2_ SNSs and MeB‐SnS_2_ MFs complexes with a microscopic area of 72 × 48 µm^2^ were collected, and the corresponding Raman spectra, Raman mapping and scatter distribution were shown Figure 4C,F and Figure S11. The relative standard deviation (RSDs) of SnS_2_ SNSs and SnS_2_ MFs for 10^−9^ M MeB molecules at Raman shifts of 1617 cm^−1^ is as low as 5.45% and 3.56%, respectively, suggesting the extremely excellent SERS enhanced uniformity. Furthermore, the stability of SERS enhancement for SnS_2_ SNSs, SnS_2_ MSs, SnS_2_ MFs substrates was proved by detecting the Raman spectra of 10^−7^ M MeB on SnS_2_ nanostructures stored for 5 months (Figure S12). Compared with the Raman intensity of MeB on these three fresh SnS_2_ nanostructures, the average Raman intensity of MeB on SnS_2_ SNSs, SnS_2_ MSs, SnS_2_ MFs substrates after storing 5 months was only discounted 12.12%, 16.70% and 17.81%, respectively, which still maintained a significant Raman enhanced effect. Finally, other common dye molecules of R6G, methyl violet (MV) and Rhodamine B (RhB) were selected to explore the generality of enhancement for SnS_2_ nanostructures with three kinds of morphologies. Different from the SERS‐enhanced MeB molecules on these three kinds of SnS_2_ nanostructures, their optimal excitation wavelengths for MV and RhB molecules are both 633 nm and R6G molecules are 532 nm (Figure S13A,C,E). Moreover, it can be clearly identified from Figure S13B,D,F and Figure 4H that SnS_2_ SNSs, SnS_2_ MSs, SnS_2_ MFs substrates display different degrees SERS enhancement on MV, R6G and RhB molecules, indicating the excellent SERS enhanced generality. With respect to 10^−7^ M MeB molecules, SnS_2_ SNSs represent the best SERS enhancement. In terms of 10^−7^ M MV, R6G and RhB molecules, SnS_2_ MSs exhibit stronger SERS enhancement, while SnS_2_ SNSs show the weakest SERS enhancement. In conclusion, such extraordinary uniformity, generality, stability, and ultra‐high sensitivity of SERS enhancement can stimulate SnS_2_ nanostructures to exhibit a promising application prospect in practical SERS detection.

### 3.5 SERS Enhanced Mechanism of SnS_2_ Nanostructures With Three Kinds of Morphologies

**FIGURE 5:**
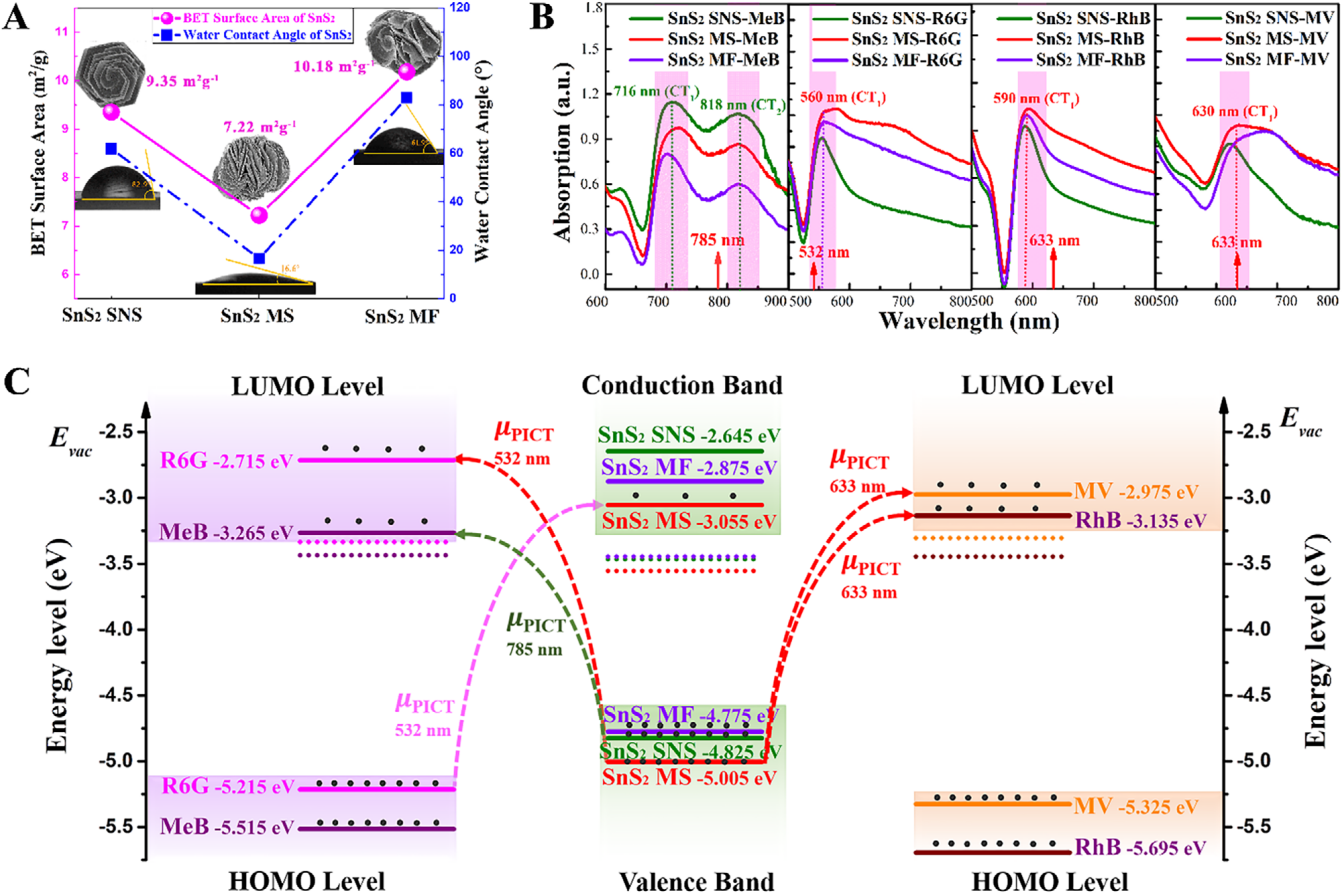
Research on the SERS enhancement mechanism of SnS_2_ nanostructures with three kinds of morphologies. (A) Hydrophilic angle and specific surface area BET of SnS_2_ SNSs, SnS_2_ MSs, and SnS_2_ MFs. (B) The charge‐transfer transitions obtained by subtracting the absorption spectrum of molecules from that of 10^−7^ M MeB, MV, R6G and RhB on SnS_2_ SNSs, SnS_2_ MSs, SnS_2_ MFs. (C) The energy level distributions of MeB, MV, R6G, RhB powder, and SnS_2_ SNSs, SnS_2_ MSs, SnS_2_ MFs.

With respect to the SERS‐enhanced mechanism of semiconductor substrates, the contribution of CM caused by PICT resonance, EM originated from surface plamon resonance (SPR) and molecular physical adsorption regulated by the morphology of nanomaterials are generally considered. First, the influence of the morphology for SnS_2_ nanostructures on SERS performance was explored by measuring the hydrophilic angle and specific surface area of SnS_2_ SNSs, SnS_2_ MSs, SnS_2_ MFs. As shown in Figure 5A, SnS_2_ SNSs and SnS_2_ MFs do not have hydrophilic properties, while SnS_2_ MSs exhibit good hydrophilicity, which induces the capillary effect on the surface of SnS_2_ MSs, thus leading to the generation of the physical enrichment for the low concentration of molecules on SnS_2_ MSs [20]. Additionally, the specific surface areas of SnS_2_ nanostructure with three kinds of morphologies are all relatively small of 7.22, 9.35, and 10.18 m^2^g^−1^, respectively, indicating the extremely weak influence on SERS performance of SnS_2_ nanostructures controlled by the physical absorption specific surface area. According to the UV–vis absorption spectra of SnS_2_ SNSs, SnS_2_ MSs and SnS_2_ MFs (Figure S14), SnS_2_ nanostructures have no obvious SPR absorption peak in the visible region, which indicates EM enhancement have almost no contribution to the SERS performance of SnS_2_ nanostructures. Therefore, CM enhancement dominated by the PICT is considered as the main SERS enhancement mechanism of SnS_2_ nanostructure with three kinds of morphologies. Therefore, the UV–vis optical absorption spectra of 10^−7^ M MeB, MV, R6G and RhB on three kinds of SnS_2_ nanostructures (Figure S15) were measured to obtain the charge‐transfer transitions (Figure 5B). It is found that the strongest charge transfers of MeB occur at 716 and 818 nm on SnS_2_ SNSs, which is much susceptible to the optimal excitation wavelength of 785 nm for MeB molecules on SnS_2_ nanostructures and the highest SERS enhancement of MeB on SnS_2_ SNSs. By analogy, the strongest charge transfers between R6G, RhB, MV and SnS_2_ MSs locate at 560, 590, and 630 nm, which are close matching to the optimal incident lasers of 532, 633, and 633 nm, respectively, and correspond to the strongest SERS enhancement of R6G, RhB, MV on SnS_2_ MSs. It is worth noting that the charge transfers between MeB, R6G, RhB, MV molecules and three kinds of SnS_2_ nanostructures can be demonstrated by the overall movement of the Sn*3*d_5/2_ and Sn*3*d_3/2_ XPS peaks of molecule‐SnS_2_ nanostructure complexes toward the lower binding energy relative to the XPS spectra of SnS_2_ nanostructures (Figure S16). Therefore, the CM enhancement attributed by PICT apparently is the dominant contribution for these three kinds of SnS_2_ nanostructures.

Further, the energy band structures of SnS_2_ nanostructure with three kinds of morphologies and MeB, R6G, RhB, MV molecules were experimentally determined by UV–vis optical absorption spectra, Mott–Schottky plots and valence band XPS spectra (Figures S14, S17–S19). The band gaps ($E_{g}$) of SnS_2_ SNSs, SnS_2_ MSs, SnS_2_ MFs powder were revealed as 2.18, 1.95, and 1.90 eV, respectively. The valence band maximum (VBM) energy $E_{\mathrm{VB}}$ is obtained by extrapolating from the emission edge of valence band XPS spectra to confirm the exact energy band position. As shown in Figure S18, SnS_2_ SNSs, SnS_2_ MSs, SnS_2_ MFs display VBs with edge of the maximum energy at about 1.36, 1.45, and 1.33 eV, and the VBM energy of MeB, R6G, RhB, MV molecules is located at 2.08, 1.88, 2.25, and 2.02 eV, respectively. The flat‐band potentials ($V_{\mathrm{fb}}$) are obtained by extrapolating the curves of Mott–Schottky plots to the *x*‐axis, which can be used to obtain the Fermi levels in the vacuum $E_{F,\mathrm{vac}}$ based on the follow formula:

$$E_{F,\mathrm{vac}}=e\left(-4.5-V_{\mathrm{fb}}-0.205\right)eV$$

The VBM energy in the vacuum $E_{\mathrm{VB},\mathrm{vac}}$ is determined by the $E_{\mathrm{VB}}$ and $E_{F,\mathrm{vac}}$, further the conduction band energy in the vacuum $E_{\mathrm{CB},\mathrm{vac}}$ is determined by the $E_{\mathrm{VB},vac}$ and $E_{g}$. The values of $E_{\mathrm{VB},\mathrm{vac}}$ and $E_{\mathrm{CB},\mathrm{vac}}$ for SnS_2_ SNSs, SnS_2_ MSs, SnS_2_ MFs and MeB, R6G, RhB, MV molecules are shown in Table S2. Based on the above analysis, the preliminary energy level positions of SnS_2_ nanostructure with three kinds of morphologies and molecules are experimentally determined (Figure 5C). It is worthwhile to note that although the developed SnS_2_ nanostructures with three kinds of morphologies in this research exhibit the same crystal structure, but also have different band structures. It is because the morphology engineering leads to the generation of lattice strain in SnS_2_ MSs and the decrease of crystallinity for SnS_2_ MFs, thus regulating the band structure of SnS_2_ nanostructures. According to Figure 5C, we can observe that the energy level differences of the electron transitions from the valence bands of SnS_2_ SNSs, SnS_2_ MSs, SnS_2_ MFs to the LUMO level of MeB molecules are 1.56, 1.74, and 1.51 eV, respectively. Among them, the energy level difference of SnS_2_ SNSs‐MeB complexes is closer matching to the energy of 1.58 eV for the 785 nm excitation laser, indicating that the strongest PICT resonance can occur on the SnS_2_ SNSs‐MeB complexes to obtain the highest SERS enhanced effect under the excitation laser of 785 nm, which is consistent with the optimal excitation wavelengths and SERS enhancement obtained by the experimental Raman spectra. With respect to the R6G, RhB and MV molecules, the energy level differences of the electron transition from valence bands of SnS_2_ MSs to LUMO levels of molecules are 2.29, 1.87, and 2.03 eV, respectively, which are much matched with the energy of 2.33 and 1.96 eV for the 532 and 633 nm excitation lasers. It is indicated that SnS_2_ MSs‐R6G complexes can produce a strong PICT resonance under the excitation laser of 532 nm, while the SERS enhancement effect of RhB and MV molecules on SnS_2_ MSs is significantly better than that of other substrates of SnS_2_ SNSs and SnS_2_ MFs due to the generation of PICT resonance with the excitation laser of 633 nm. Therefore, it is reasonable that the SERS enhancement mechanism of SnS_2_ nanostructure with three kinds of morphologies to MeB, R6G, RhB, MV molecules is derived from the dominant contribution of PICT resonance with different wavelength excitation lasers.

## 4 Conclusions

In summary, in the face of the problem that the SERS sensitivity of pure semiconductors is generally lower than that of noble metals, ultra‐sensitive SnS_2_ nanostrctures with three kinds of morphologies were developed. Based on DFT calculations, 2D SnS_2_ with better SERS performance for MeB molecules was screened out from Sulfides and Selenides. Through adjusting the concentration of reactants to control the growth driving force without any surfactants or templates, SnS_2_ SNSs, SnS_2_ MSs and SnS_2_ MFs were successfully synthesized, which all exhibit ultra‐low LODs of 10^−12^, 10^−13^, and 10^−11^ M, as well as high EFs of 4.3 × 10^8^, 1.6 × 10^7^, and 3.0 × 10^8^, respectively. To the best of our knowledge, it is one of the highest sensitivities among the reported pure semiconductor substrates and even can parallel to the noble metal substrates with “hot spot” effect. This extraordinary SERS enhancement of SnS_2_ nanostructures with three kinds of morphologies to different molecules can be attributed to the PICT resonance with different wavelength excitation lasers based on the experimental energy band structures. Furthermore, SnS_2_ SNSs, SnS_2_ MSs and SnS_2_ MFs all exhibit excellent uniformity, generality, and stability of the SERS enhancement, which shows broad prospects in the practical application of SERS technology. Benefitting to the excellent SERS performance of 2D SnS_2_ materials and the advantages that the PICT resonance enhancement excited for different probe molecules is not limited by its morphology, it is expected to provide a class of potential commercial SERS active materials for the practical application of semiconductor‐based SERS technology, including the detection fields of volatile organic compounds VOCs, heavy metal ions, antibiotic, pesticide residue, protein and MicroRNA and other tumor markers [28].

## Conflicts of Interest

The authors declare no conflicts of interest.

## Supporting information



Supporting Information

## Data Availability

The raw data and processed data required to reproduce these findings are available from the corresponding author upon request.
