# Supplementary material for: Morphology Engineering of SnS2 Nanostructures to Stimulate PICT Resonance for Ultra‐Sensitive SERS Sensors
Source: Exploration (Beijing). 2025 Feb 24;5(3):270016. doi: 10.1002/EXP.70016 (PMC12199321; doi:10.1002/EXP.70016)
Supplement: Supplementary file 1 — Supporting Information [file EXP2-5-270016-s001.docx]

Supporting Information

**Morphology engineering of SnS_2_ nanostructures to stimulate PICT resonance for ultra-sensitive SERS sensors**

Yusi Peng ^1,2^, Weida Zhang ^1,2,3^, Meimei Xu ^1,2,3^, Shuai Zhao ^1,2,3^, Lili Yang^4^, Dan Li ^1,2,3^, Masaki Tanemura ^5^, Zhengren Huang ^1^*, Yong Yang ^1,2^*

^1^ State Key Laboratory of High-Performance Ceramics and Superfine Microstructures, Shanghai Institute of Ceramics, Chinese Academy of Sciences, 1295 Dingxi Road, Shanghai 200050, People’s Republic of China.

^2^ University of Chinese Academy of Sciences, No.19(A) Yuquan Road, Beijing 100049, People’s Republic of China.

^3^ Center of Materials Science and Optoelectronics Engineering, University of Chinese Academy of Sciences, Beijing 100049, China.

^4^ College of Integrated Circuit Science and Engineering, Nanjing University of Posts and Telecommunications, Nanjing 210023, China.

^5^ Department of Frontier Materials, Graduate School of Engineering, Nagoya Institute of Technology, Showa, Nagoya, 466-8555, Japan.

*Corresponding Author. E-mail: [yangyong@mail.sic.ac.cn](mailto:yangyong@mail.sic.ac.cn);

zhrhuang@mail.sic.ac.cn;

**Enhancement factor (EF) calculations**

SERS enhancement factors (EFs) of SnS_2_ SNSs, SnS_2_ MSs and SnS_2_ MFs substrates for MeB molecules were calculated by the following general formula ^[1]^:

$\text{EF=}\frac{\text{I}_{\text{SERS}}}{\text{I}_{\text{prob}}}\text{×}\frac{\text{N}_{\text{prob}}}{\text{N}_{\text{SERS}}}$ (1)

Based on formula (1), *I_SERS_* and *I_prob_* are the Raman intensity at a selected Raman peak of molecule-semiconductor complex and MeB probe molecules. *N_SERS_* is the average number of MeB molecules on SnS_2_ nanostructure with three morphologies in the Raman detection region. *N_prob_* is the the average number of MeB powder in the Raman detection region. With respect to the average number of MeB molecules SERS enhanced by SnS_2_ nanostructure substrates$N_{SERS-1}$:

$N_{SERS-1}=\frac{C_{sol}V_{sol}N_{A}A_{Raman}}{A_{substrate}}$ (2)

As for the average number of MeB molecules SERS enhanced by SnS_2_ MS substrates with molecular physical enrichment of 40 times $N_{SERS-2}$:

$N_{SERS-2}=40{\times N}_{SERS-1}=\frac{40{\times C}_{sol}V_{sol}N_{A}A_{Raman}}{A_{substrate}}$ (3)

As for the average molecular number of MeB powder without SERS enhancement $N_{prob}$:

$N_{prob}=C_{prob}hN_{A}A_{Raman}$ (4)

where $C_{sol}$ (molL^-1^) is the concentration of the detected MeB molecules. $V_{sol}$ (L) is the volume of the mixture solution of SnS_2_ nanostructure powder and detected MeB molecules that finally dropped on the glass slide, which is about 5 μL. $A_{Raman}$ and $A_{substrate}$ (m^2^) are the laser radiation area and the droplet diffusion area of SnS_2_ microspheres powder mixture on the glass slide, respectively. And the circle diameter of this droplet diffusion is about 0.8 cm after measurement. $\text{C}_{\text{prob}}$ of MeB powder is 3.13 M. $h$ is the laser radiation depth, which is about 21 μm ^[1]^.

As for the SERS enhanced condition of MeB molecules on SnS_2_ MS substrates with molecular physical enrichment of 40 times:

$\frac{N_{prob}}{N_{SERS-2}}=\frac{C_{prob}hN_{A}A_{Raman}A_{substrate}}{{40\times C}_{sol}V_{sol}N_{A}A_{Raman}}=\frac{C_{prob}hA_{substrate}}{C_{sol}V_{sol}}$

$=\frac{3.13\times2.1\times{10}^{-5}\times\pi\times0.16\times{10}^{-4}}{40\times5\times{10}^{-6}\times C_{sol}}=\frac{1.66\times{10}^{-5}}{C_{sol}}$ (5)

As for the SERS enhanced condition of MeB molecules on SnS_2_ SNSs and SnS_2_ MFs without molecular physical enrichment:

$\frac{N_{prob}}{N_{SERS-1}}=\frac{C_{prob}hN_{A}A_{Raman}A_{substrate}}{C_{sol}V_{sol}N_{A}A_{Raman}}=\frac{C_{prob}hA_{substrate}}{C_{sol}V_{sol}}$

$=\frac{3.13\times2.1\times{10}^{-5}\times\pi\times0.16\times{10}^{-4}}{5\times{10}^{-6}\times C_{sol}}=\frac{6.64\times{10}^{-4}}{C_{sol}}$ (6)

Under the excitation laser of 785 nm, the Raman intensity $\text{I}_{\text{prob}}$ of MeB powder at 1620 cm^-1^ is 201710.8. With respect to the SERS enhancement of 10^-13^ M MeB molecules on SnS_2_ MS substrates, the *I_SERS_* is 363575.4. Therefore,

$\text{EF= }\frac{\text{I}_{\text{SERS}}}{\text{I}_{\text{prob}}}\text{×}\frac{\text{N}_{\text{prob}}}{\text{N}_{\text{SERS}}}=\frac{\text{363575.4}}{\text{201710.8}}\text{×}1.66\times{10}^{8}\text{=}3.0\times{10}^{8}$ (7)

As for the SERS enhancement of 10^-12^ M MeB molecules on SnS_2_ SNSs substrates, the *I_SERS_* is 130625.9. Therefore,

$\text{EF= }\frac{\text{I}_{\text{SERS}}}{\text{I}_{\text{prob}}}\text{×}\frac{\text{N}_{\text{prob}}}{\text{N}_{\text{SERS}}}=\frac{130625.9}{\text{201710.8}}\text{×}\frac{6.64\times{10}^{-4}}{{10}^{-12}}\text{=4.3}\times{10}^{8}$ (8)

As for the SERS enhancement of 10^-11^ M MeB molecules on SnS_2_ MFs substrates, the I*_SERS_* is 48605.1. Therefore,

$\text{EF= }\frac{\text{I}_{\text{SERS}}}{\text{I}_{\text{prob}}}\text{×}\frac{\text{N}_{\text{prob}}}{\text{N}_{\text{SERS}}}=\frac{48605.1}{\text{201710.8}}\text{×}\frac{6.64\times{10}^{-4}}{{10}^{-11}}\text{==1.6}\times{10}^{7}$ (9)

**Table S1**. Reported enhancement factors on different nanostructure SERS substrates.

| **SERS substrates** | | **Probe molecules** | **Excitation wavelength** | **EFs** | **LODs** | **Ref** |
| --- | --- | --- | --- | --- | --- | --- |
| **Nobel metal substrates** | Au nanospheres arrays | 4-ABT | 785 nm | 6.4×10^4^ | / | [2] |
|  | Ag layer@ SiO_2_ spheres | R6G | 532 nm | 10^8^ | 10^-13^ M | [3] |
|  | Ag densely nanoislands | R6G | 532 nm | / | 10^-14^ M | [4] |
|  | Ag NPs on Porous Silicon | R6G | 532 nm | 10^10^ | 10^–14^ M | [5] |
|  | Ag nanospheres arrays | R6G | 514 nm | / | 10^−20^ M | [6] |
|  | Au@ZIF-67 nanostructure | thiram | 633 nm | 1.91×10^8^ | 10^−10^ M | [7] |
|  | Au/Ag nanopillars | MB | 785 nm | 8.4×10^8^ | 10^−13^ M | [8] |
|  | Au nanorod arrays | R6G | 532 nm | 10^9^ | / | [9] |
|  | PA66NP@Au arrays | R6G | 532 nm | 10^10^ | 10^−14^ M | [10] |
|  | multi-Au@Ag-Au NPs | CV | 532 nm | 3.23×10^9^ | 3.22×10^−12^M | [11] |
| **Metal-semiconductor substrates** | GANS-Au@Ag NPs | 4-MBA | / | 1.2×10^8^ | 10^−11^ M | [12] |
|  | Au-TiO_2_ nanoparticles | SARS-CoV-2 | 785 nm | / | 100 pM | [13] |
|  | Au/ND/C_3_N_4_ nanosheets | CV | 633 nm | 6.67×10^6^ | 10^-14^ M | [14] |
|  | Ag/BP nanosheets | R6G | 633 nm | 10^11^ | 10^–20^ M | [15] |
|  | Ti_3_C_2_T_x_/Au NT | CV | 785 nm | 3.6×10^9^ | 10^–12^ M | [16] |
| **Semiconductor substrates** | Monolayer WS_2(1–x)_Se_2x_ alloys | R6G | / | 1.27×10^6^ | / | [17] |
|  | Multi-layer Nb_2_C MXenes | MeB | 532 nm | 3.0×10^6^ | 10^-8^ M | [18] |
|  | Multi-layer Ta_2_C MXenes | MV | 532 nm | 1.4×10^6^ | 10^-7^ M | [18] |
|  | Multi-layer Ti_3_C_2_ MXenes | MeB | 785 nm | 3.2×10^6^ | 10^-7^ M | [19] |
|  | Monolayer of Ti_3_C_2_ MXene | R6G | 532 nm | 3.82×10^8^ | 10^-11^ M | [20] |
|  | ZnSe nanoparticles | 4-Mpy | 514.5 nm | 2×10^6^ | 10^-3^ M | [21] |
|  | Amorphous TiO_2_ nanosheets | 4-MBA | 633 nm | 1.86×10^6^ | 6×10^-6^ M | [22] |
|  | sea urchin-like W_18_O_49_ | R6G | 532.8 nm | 3.4×10^5^ | 10^-7^ M | [23] |
|  | Porous ZnO nanosheets | 4-MBA | 514.5 nm | 10^3^ | 10^-6^ M | [24] |
|  | Amorphous MoO_3_ | R6G | 532 nm | 1.8×10^7^ | 10^–8^ M | [25] |
|  | Nanosphere Cu_2_O | R6G | 514.5 nm | 8×10^5^ | 6×10^-9^ M | [26] |
|  | Mo-doping Ta_2_O_5_ nanowires | MV | 532 nm | 2.2×10^7^ | 9×10^–9^ M | [27] |
|  | Nb_2_O_5_ nanoflowers | MV | 532 nm | 7.1×10^7^ | 10^–8^ M | [28] |
|  | Amorphous Rh_3_S_6_ microbowls | R6G | 647 nm | 10^5^ | 10^-7^ M | [29] |
|  | Metal-Like H_1.68_MoO_3_ | R6G | 633 nm | 1.1×10^7^ | 10^–9^ M | [30] |
|  | Mo_2_N flexible membrane | 2,4-DCP | 532 nm | 5.2×10^7^ | 10^–11^ M | [31] |
|  | MoN nanosheets | R6G | 633 nm | 8.2×10^6^ | 10^–10^ M | [32] |
|  | NbTe_2_ nanosheets | MB | 514 nm | 5.6×10^6^ | 10^–9^ M | [33] |
|  | MoS_2_@ZnO heterojunction | MB | 514 nm | 1.2×10^6^ | 10^-12^ M | [34] |
| **In this work** | **SnS_2_ nanosheets** | **MeB** | **785** nm | **4.3×10^8^** | **10^-12^ M** |  |
|  | **SnS_2_ microspheres** | **MeB** | **785** nm | **3.0×10^8^** | **10^-13^ M** |  |
|  | **SnS_2_ microflowers** | **MeB** | **785** nm | **1.6×10^7^** | **10^-11^ M** |  |


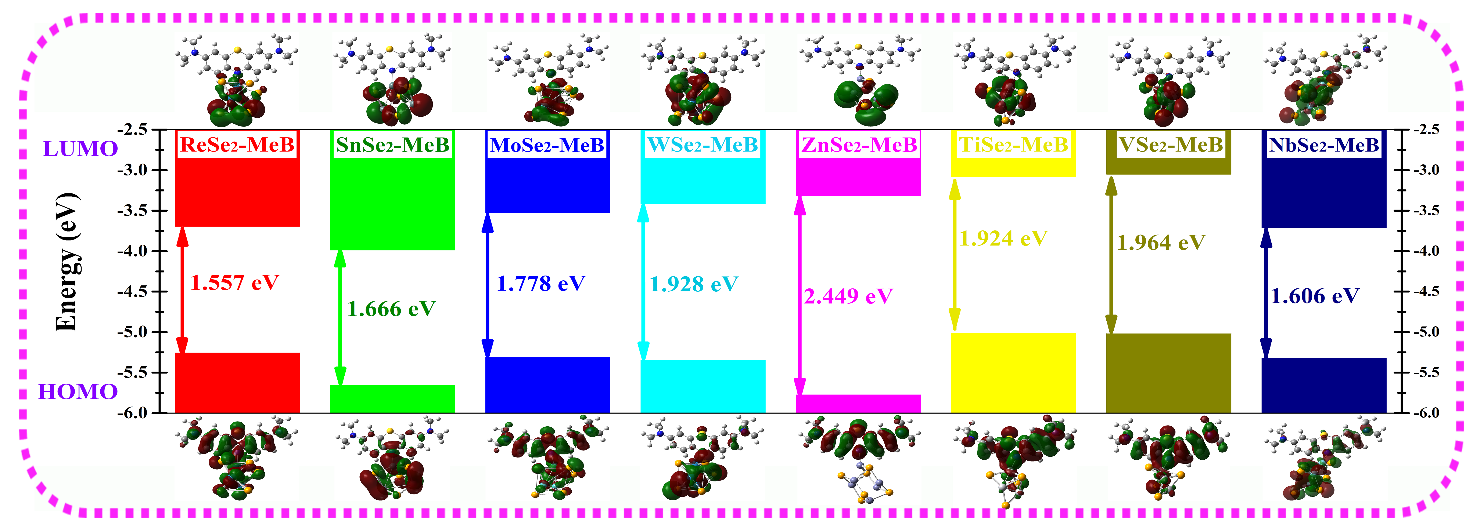


**FIGURE S1.** The energy level distributions and HOMO/LUMO illustrations of MeB-selenides.


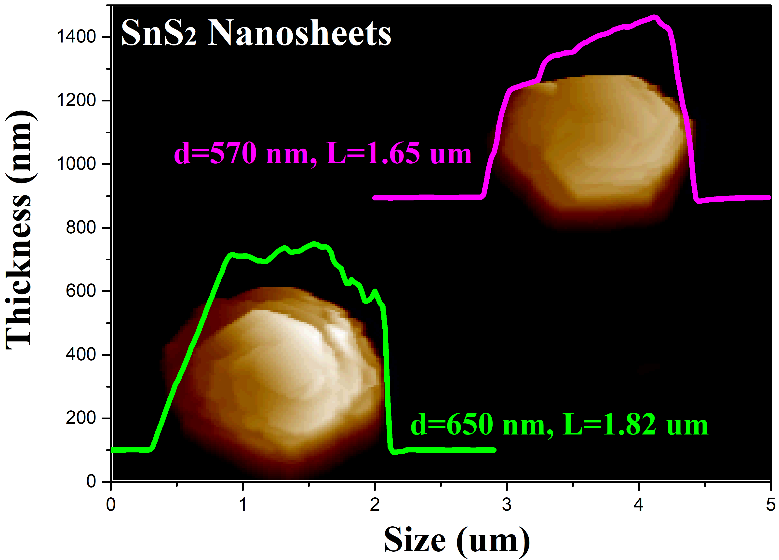


**FIGURE S2.** AFM images of SnS_2_ SNSs.


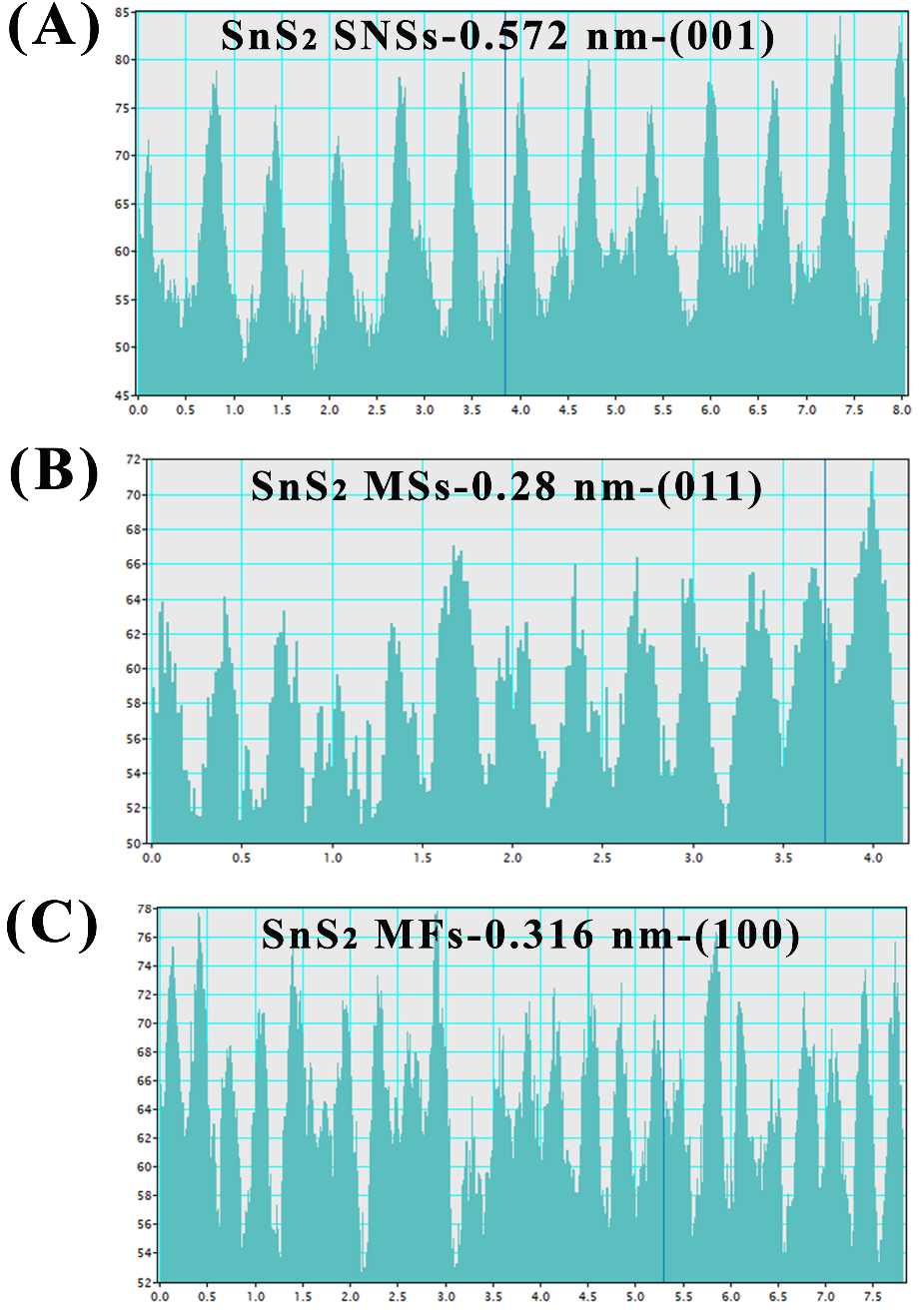


**FIGURE S3.** The lattice fringe space of HRTEM images for SnS_2_ SNSs (A), SnS_2_ MSs (B) and SnS_2_ MFs (C).


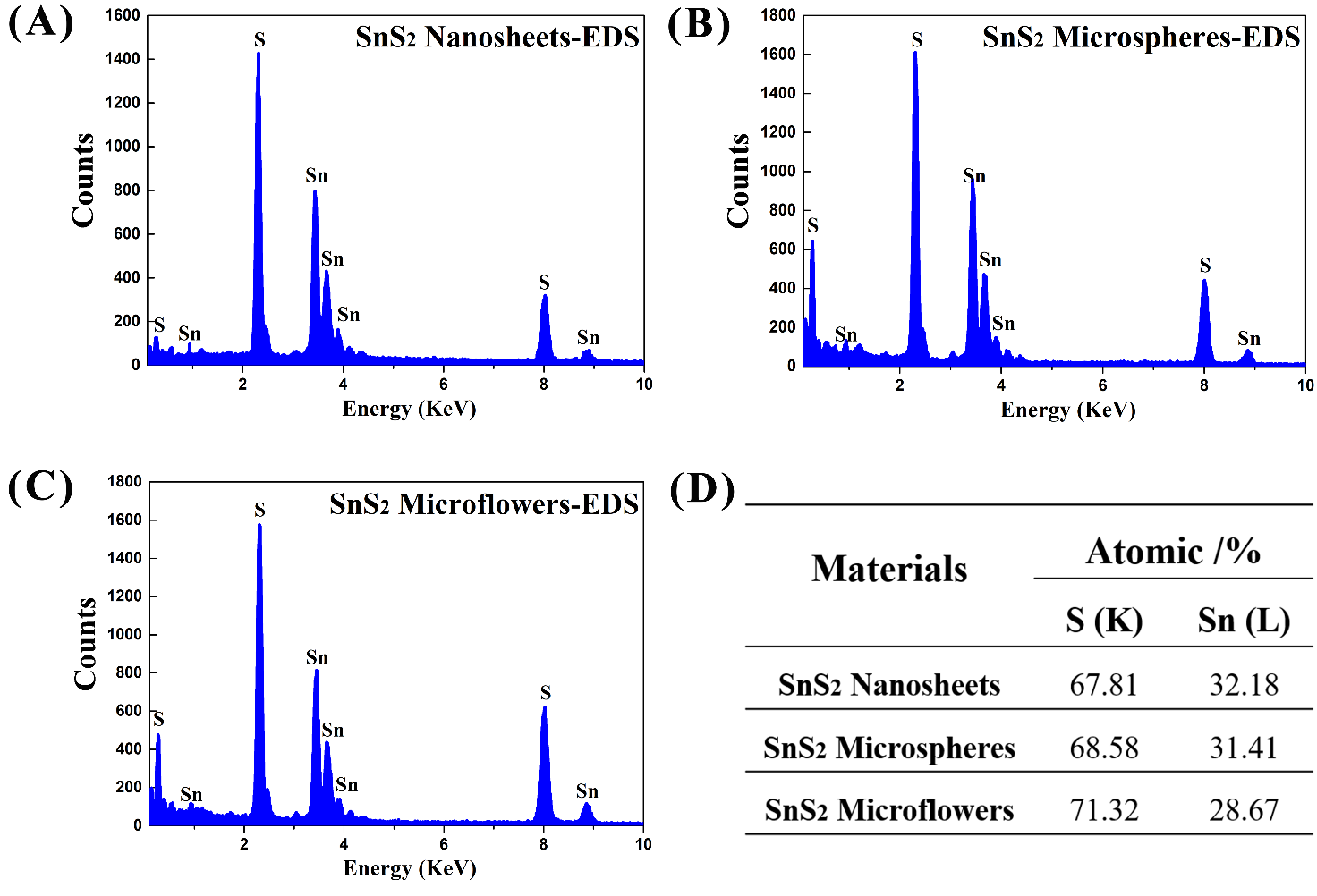


**FIGURE S4. (A-C)** EDS spectra of SnS_2_ SNSs (A), SnS_2_ MSs (B) and SnS_2_ MFs (C). **D)** Atomic ratio of Sn and S for SnS_2_ nanostructure with three morphologies.

**
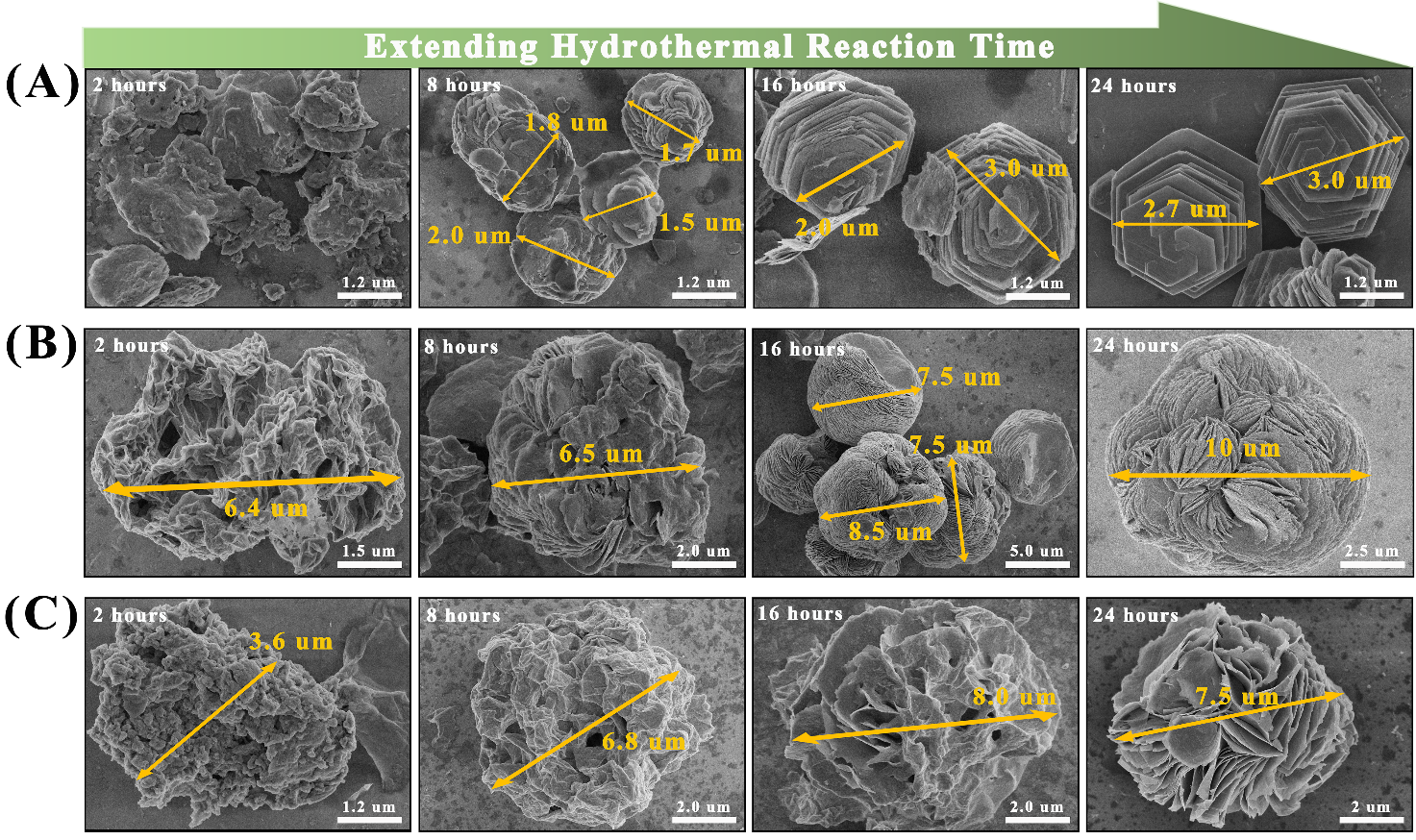
**

**FIGURE S5.** Regulating the hydrothermal reaction time to research the formation mechanism of SnS_2_ nanostructures with three morphologies. **(A-C)** SEM images of SnS_2_ SNSs (A), SnS_2_ MSs (B) and SnS_2_ MFs (C) with the hydrothermal reaction for 2 h, 8 h, 16 h, and 24 h.

**
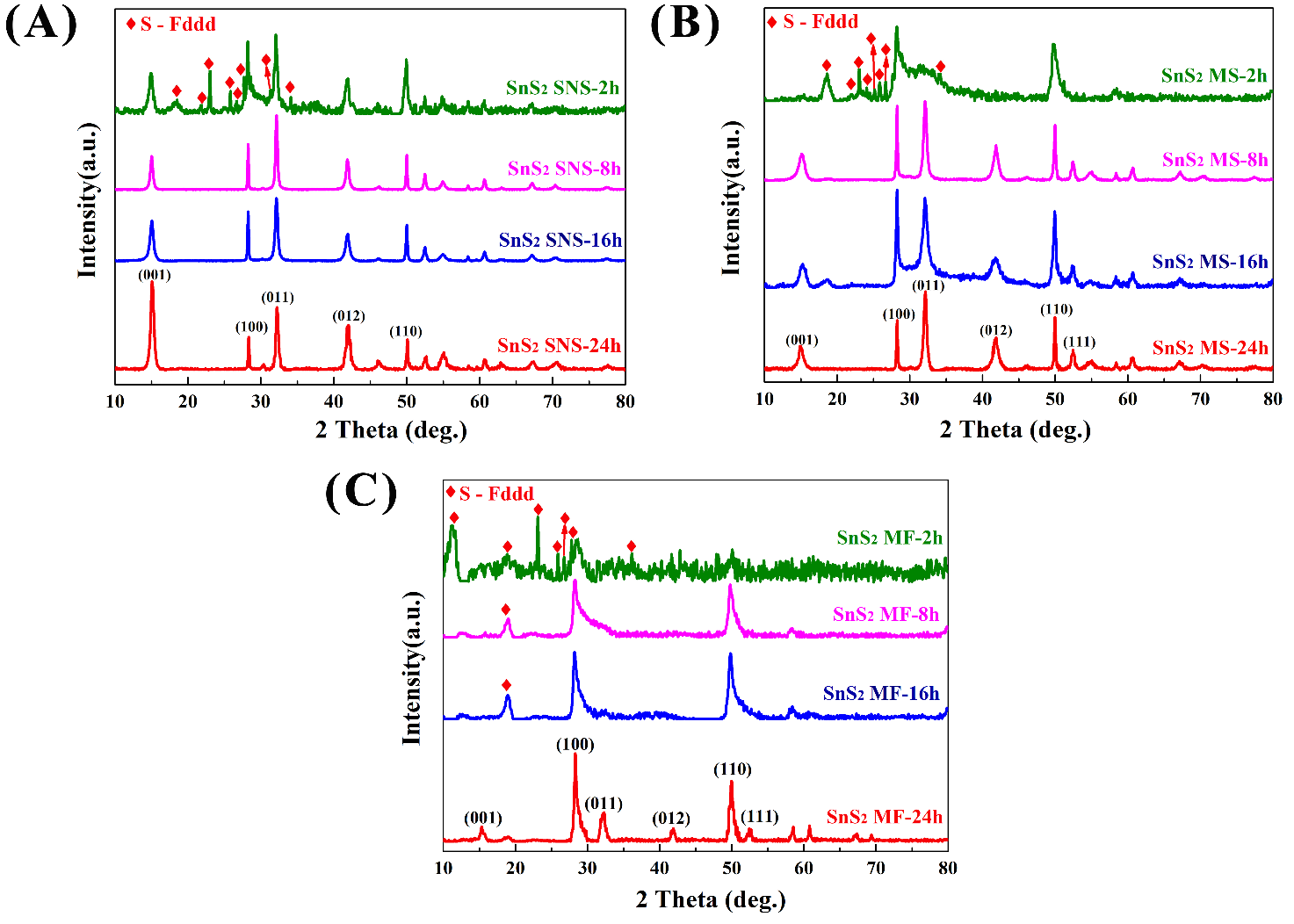
**

**FIGURE S6.** XRD spectra of SnS_2_ SNSs (A), SnS_2_ MSs (B) and SnS_2_ MFs (C) with the hydrothermal reaction for 2 h, 8 h, 16 h, and 24 h.


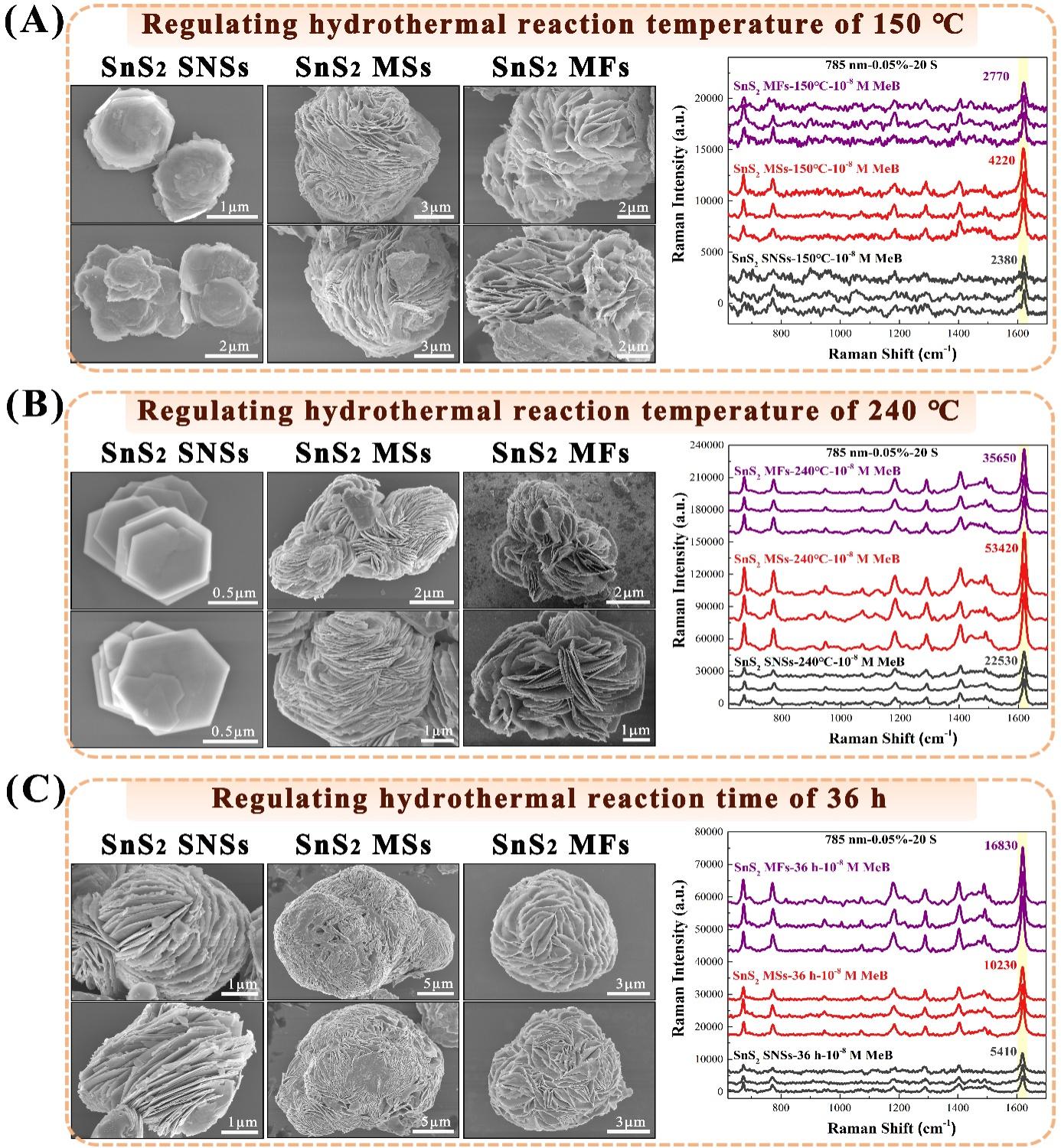


**FIGURE S7.** (A, B) SEM images and corresponding Raman spectra of 10^-8^ M MeB molecules on SnS_2_ SNSs, SnS_2_ MSs and SnS_2_ MFs with different hydrothermal reaction temperature of 150℃ (A) and 240 ℃ (B). (C) SEM images and corresponding Raman spectra of 10^-8^ M MeB molecules on SnS_2_ SNSs, SnS_2_ MSs and SnS_2_ MFs with different hydrothermal reaction time of 36 h.


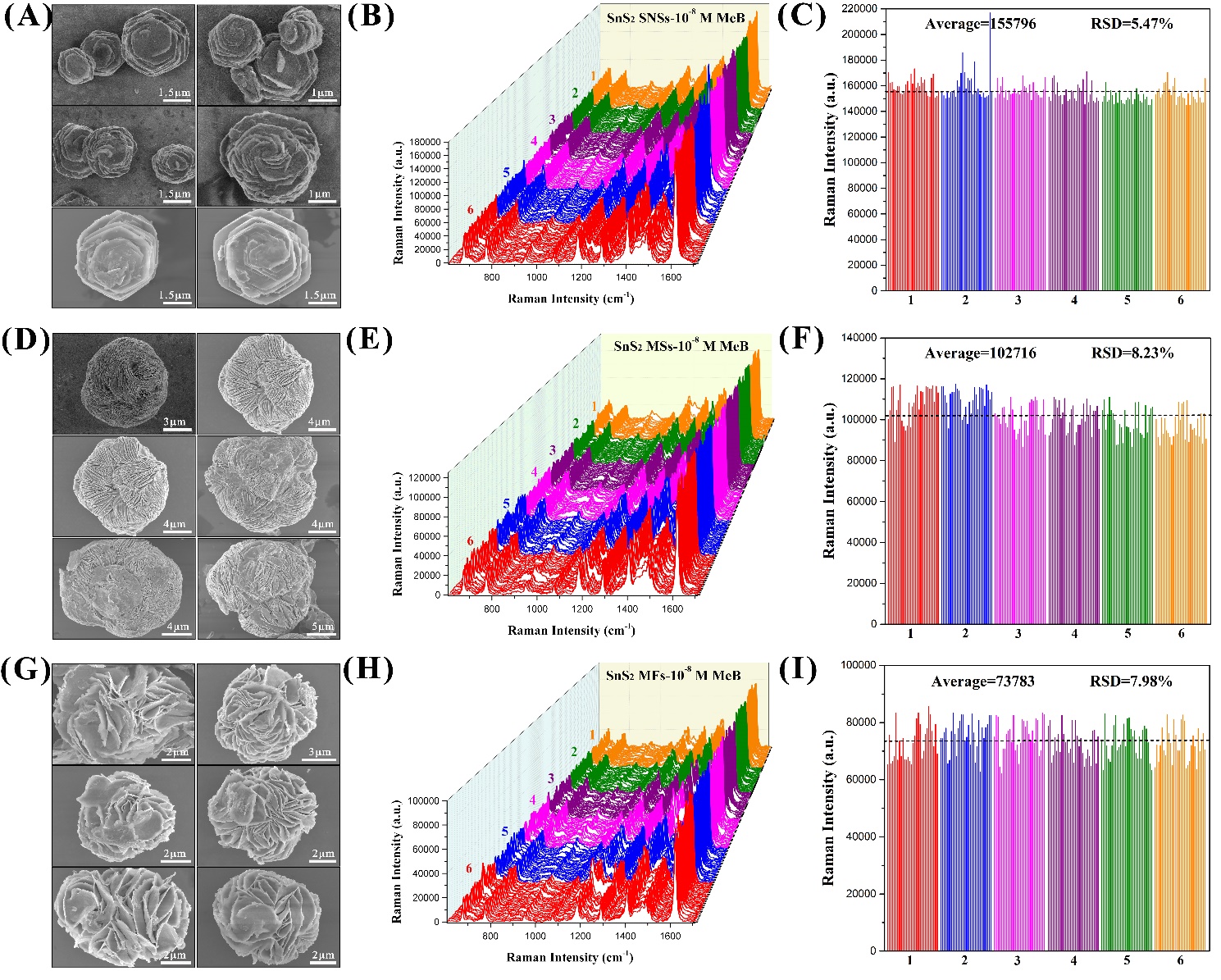


**FIGURE S8.** (A, D, G) SEM images of SnS_2_ SNSs (A), SnS_2_ MSs (D) and SnS_2_ MFs (G) with six synthesis batches. (B, E, H) Raman spectra of 10^-8^ M MeB on SnS_2_ SNSs (B), SnS_2_ MSs (E) and SnS_2_ MFs (H) with six synthesis batches. (C, F, I) The bar charts of Raman intensity at 1627 cm^-1^ for 10^-8^ M MeB on SnS_2_ SNSs (C), SnS_2_ MSs (F) and SnS_2_ MFs (I) with six synthesis batches.


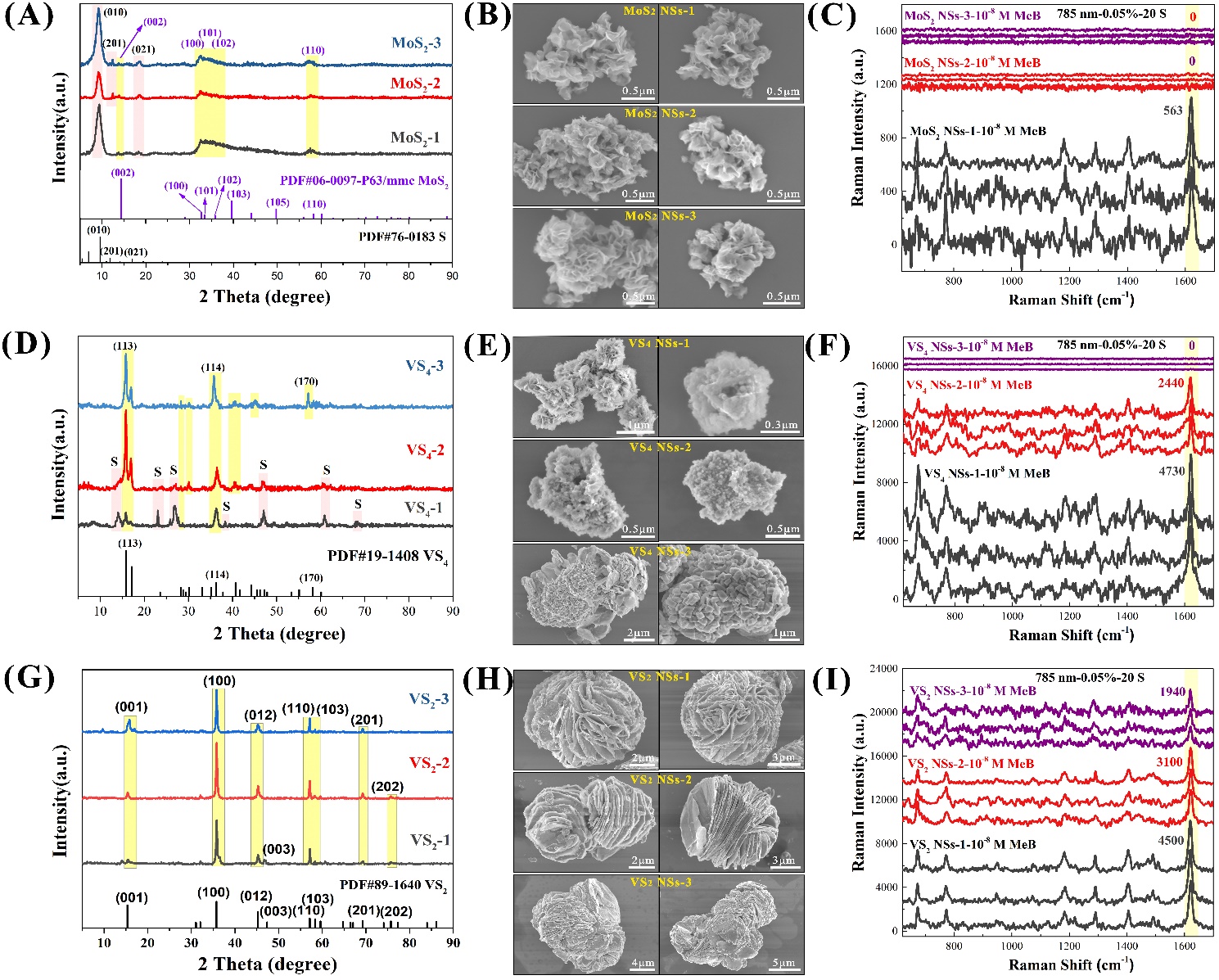


**FIGURE S9.** (A, D, G) XRD spectra of three kinds of MoS_2_ (A), VS_4_ (D) and VS_2_ (G) nanostructures. (B, E, H) SEM images of MoS_2_ (B), VS_4_ (E) and VS_2_ (H) nanostructures with three morphologies formed by regulating the concentration of reactants. (E, F, I) Corresponding Raman spectra of 10^-8^ M MeB molecules on MoS_2_ (E), VS_4_ (F) and VS_2_ (I) nanostructures with three morphologies.


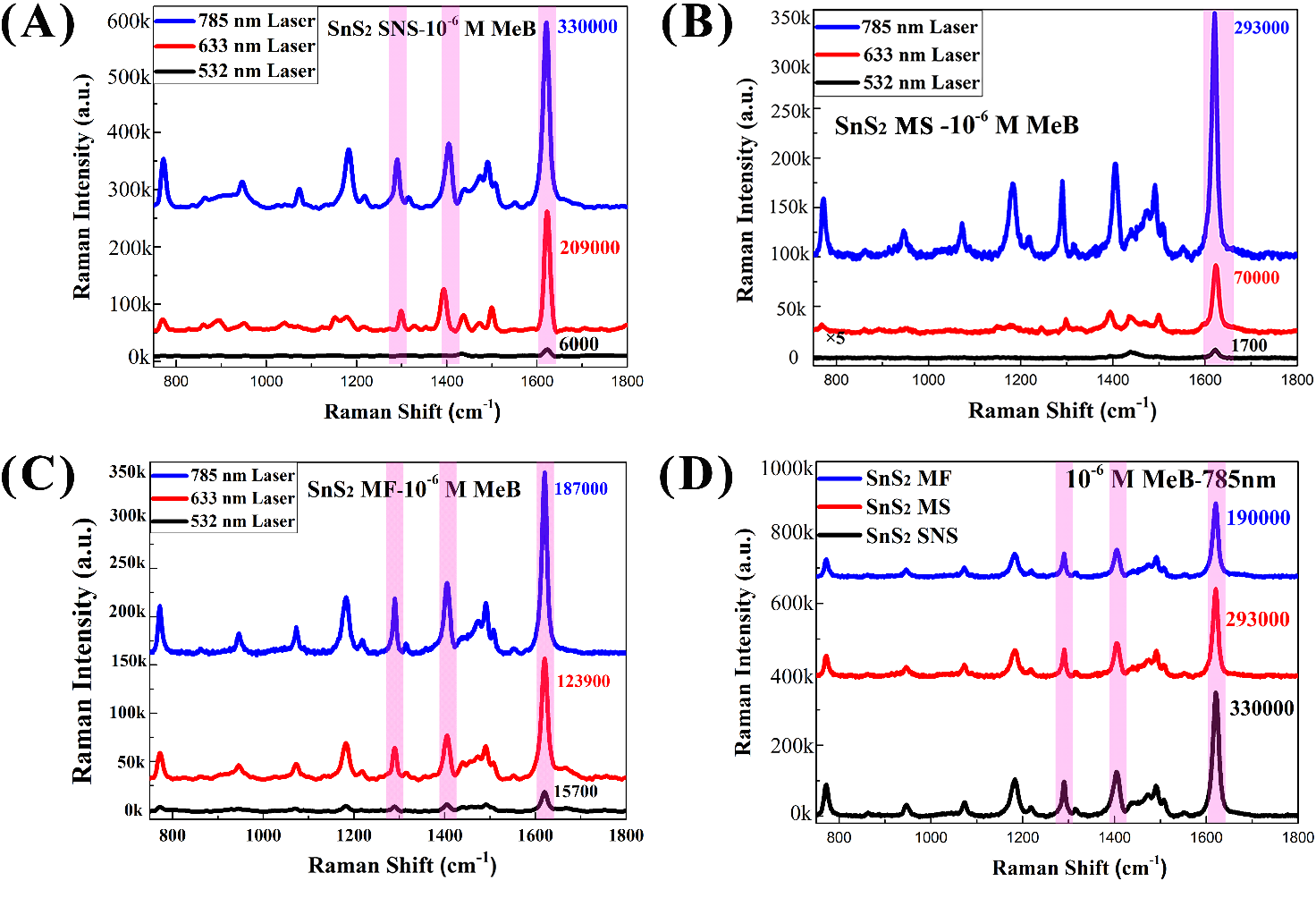


**FIGURE S10. (A-C)** Raman spectra of 10^-6^ M MeB on SnS_2_ SNSs (a), SnS_2_ MSs (b) and SnS_2_ MFs (C) under the excitation laser of 532 nm, 633 nm, and 785 nm. **(D)** Raman spectra of 10^-6^ M MeB on SnS_2_ SNSs, SnS_2_ MSs and SnS_2_ MFs under the excitation laser of 785 nm.


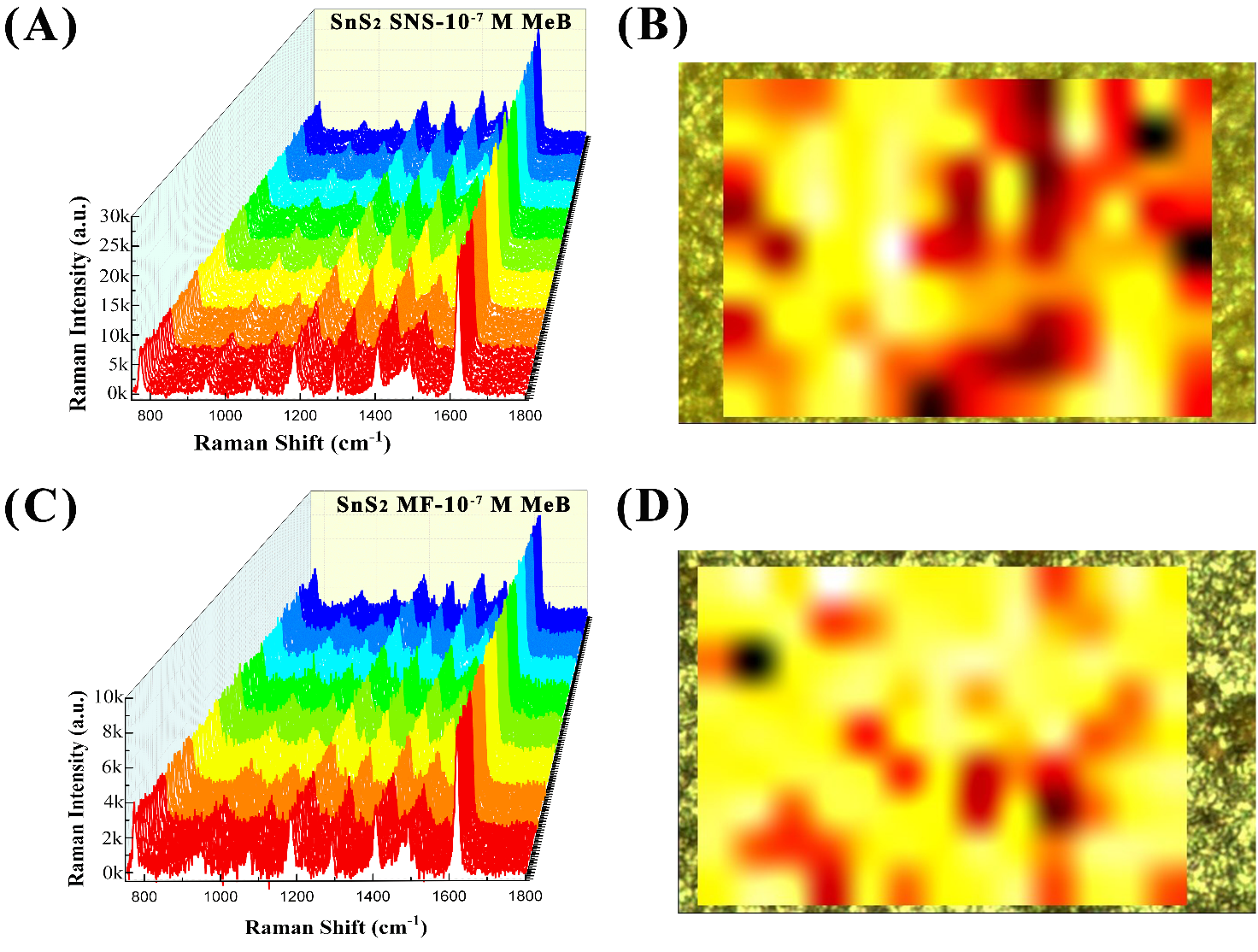


**FIGURE S11. (A, C)** Raman spectra of 118 test points in the microscope regions for SnS_2_ SNSs (A) and SnS_2_ MFs (C). **(B, D)** Raman Mapping image at 1627 cm^-1^ of 10^-9^ M MeB molecules with the area of 72$\times$48 μm^2^ on SnS_2_ SNSs (B) and SnS_2_ MFs (D) substrates.


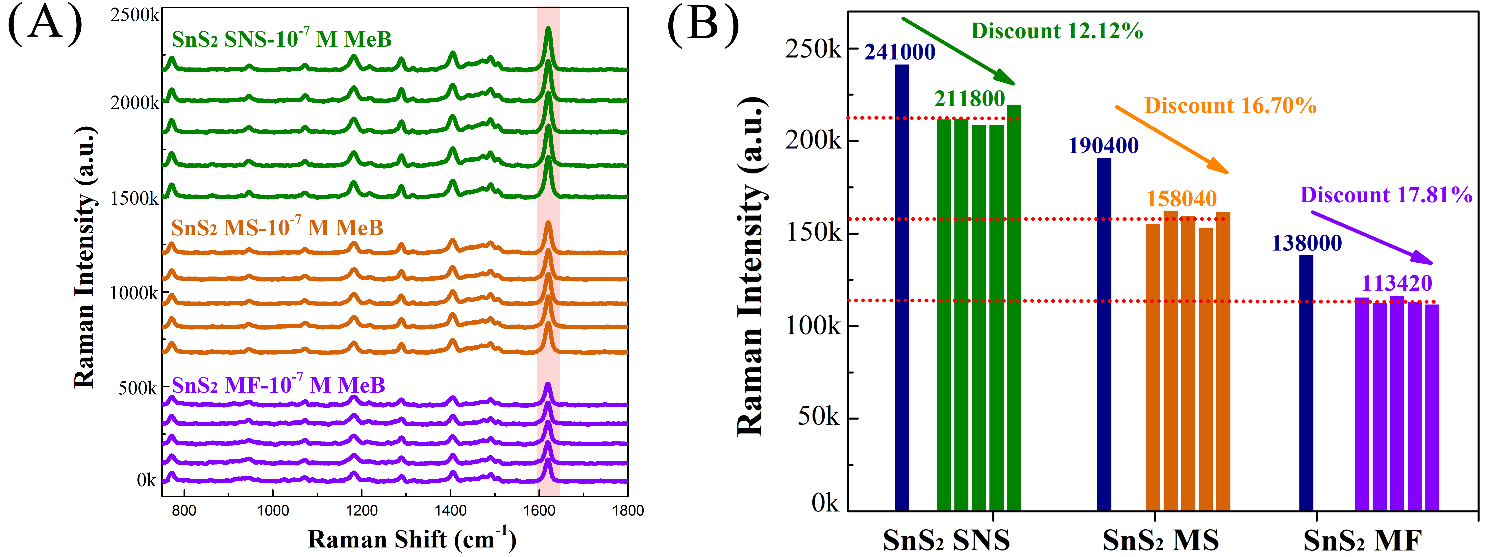


**FIGURE S12.** Research on the SERS enhanced stability of SnS_2_ nanostructures with three morphologies. (A) Raman spectra of 10^-7^ M MeB on SnS_2_ SNSs, SnS_2_ MSs, SnS_2_ MFs stored for 5 months. (B) Comparison of Raman intensity for MeB molecules on fresh SnS_2_ nanostructures and SnS_2_ nanostructures after 5 months.


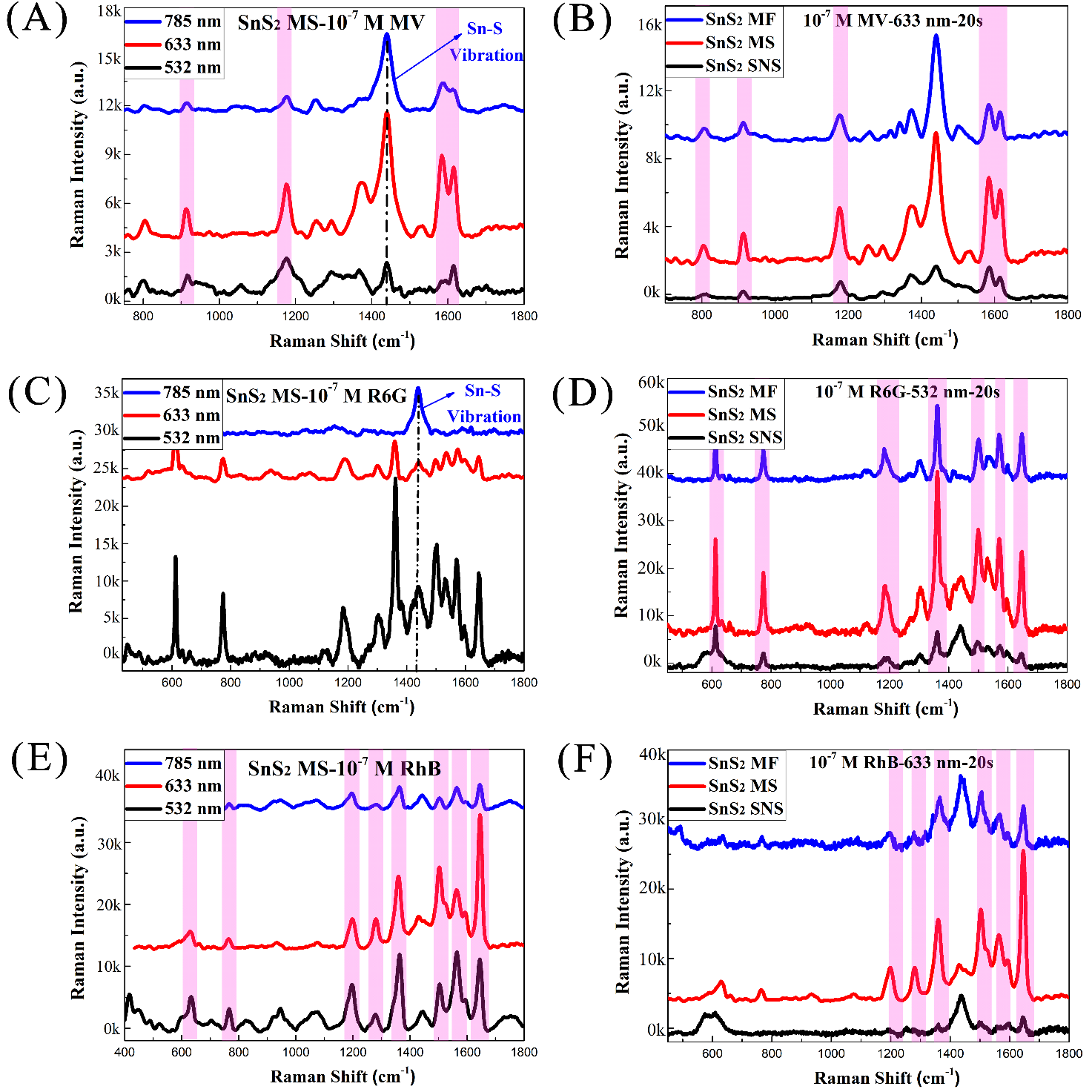


**FIGURE S13. (A, C, E)** Raman spectra of 10^-6^ M MV (A), R6G (C) and RhB (E) on SnS_2_ SNSs, SnS_2_ MSs, SnS_2_ MFs under the excitation laser of 532 nm, 633 nm, and 785 nm. **(B, D, F)** Raman spectra of 10^-7^ M MV (B), R6G (D) and RhB (F) on SnS_2_ SNSs, SnS_2_ MSs, SnS_2_ MFs.


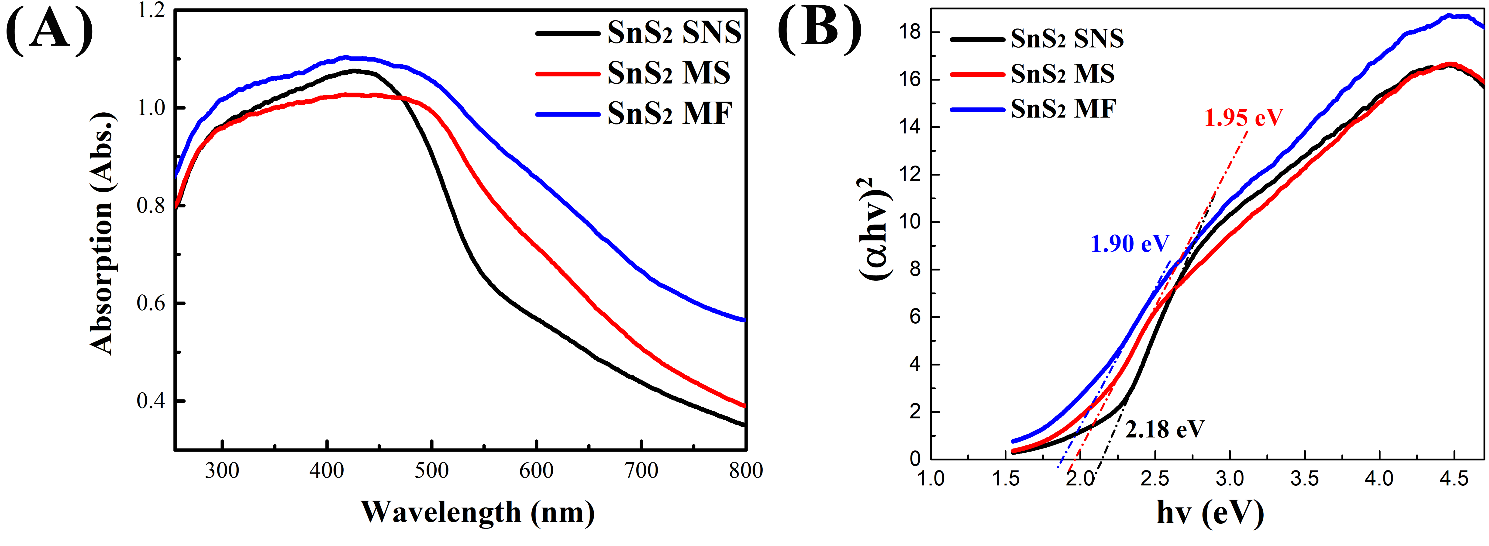


**FIGURE S14. (A)** UV-Vis optical absorption spectrum of SnS_2_ SNSs, SnS_2_ MSs and SnS_2_ MFs. **(B)** Plot of ${(\alpha hv)}^{2}$ as a function of photon energy, Eg, for SnS_2_ SNSs, SnS_2_ MSs and SnS_2_ MFs.


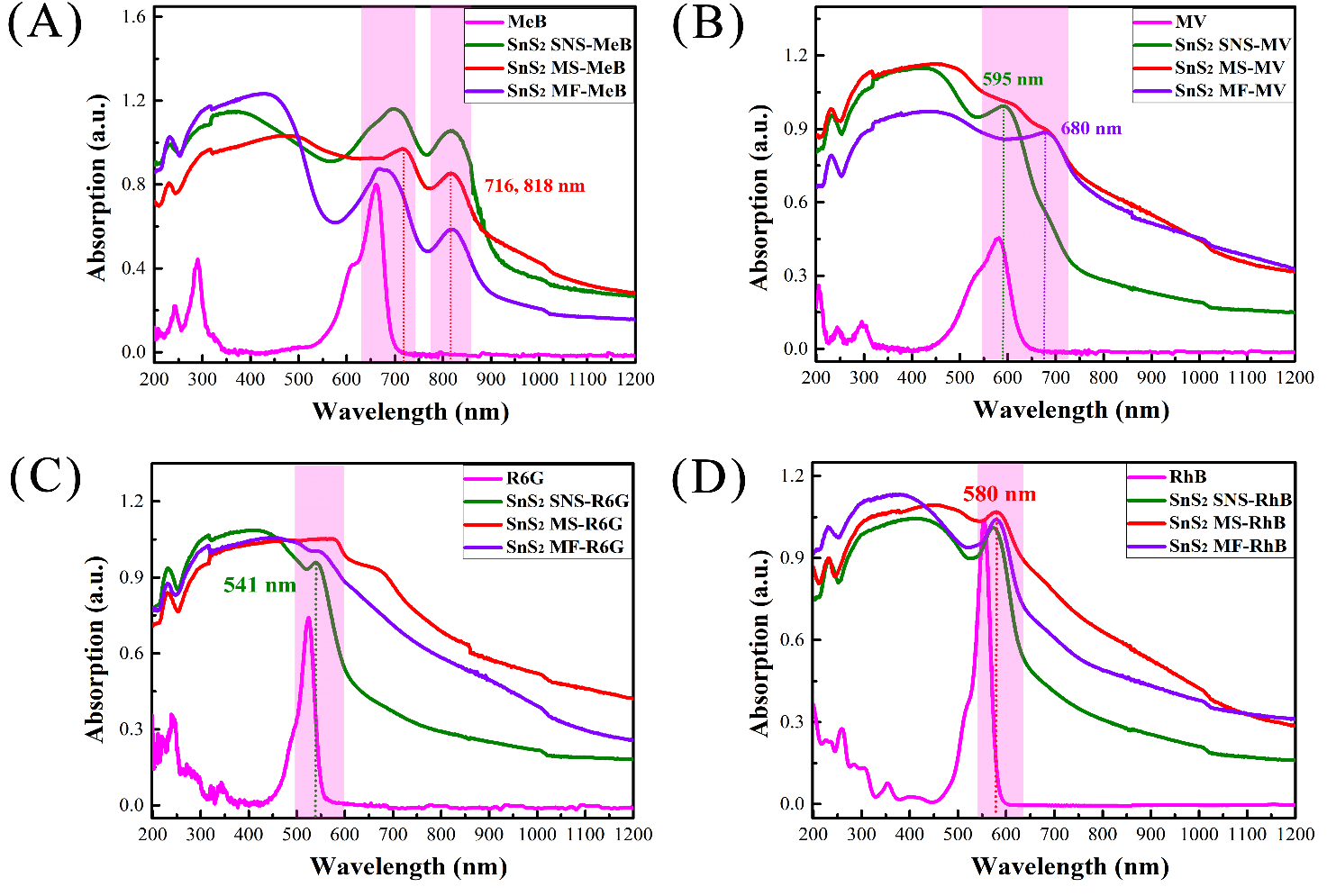


**FIGURE S15. (A-D)** UV-Vis optical absorption spectra of 10^-7^ M MeB (A), MV (B), R6G (C) and RhB (D) on SnS_2_ SNSs, SnS_2_ MSs, SnS_2_ MFs.


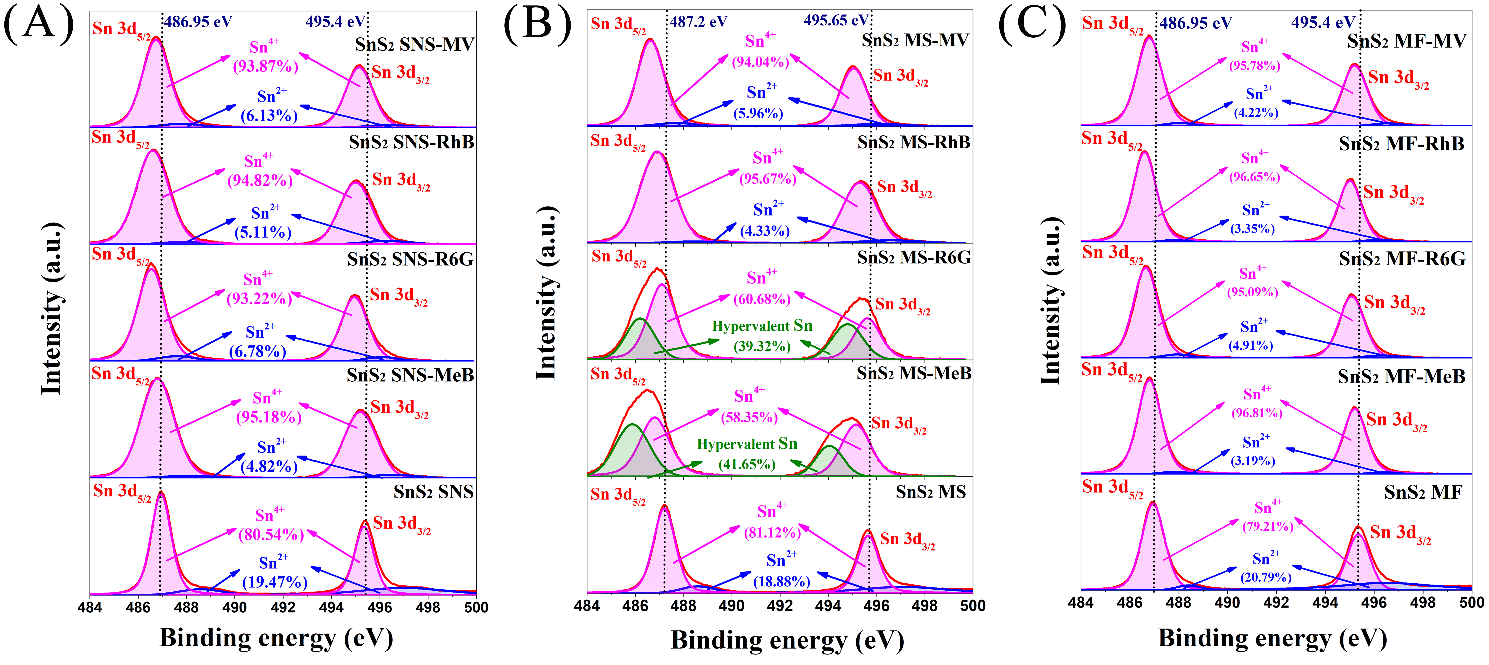


**FIGURE S16. (A-C)** Sn*3d* XPS spectrum of MeB, MV, R6G and RhB molecules on SnS_2_ SNSs **(A)**, SnS_2_ MSs **(B)**, SnS_2_ MFs **(C)**.


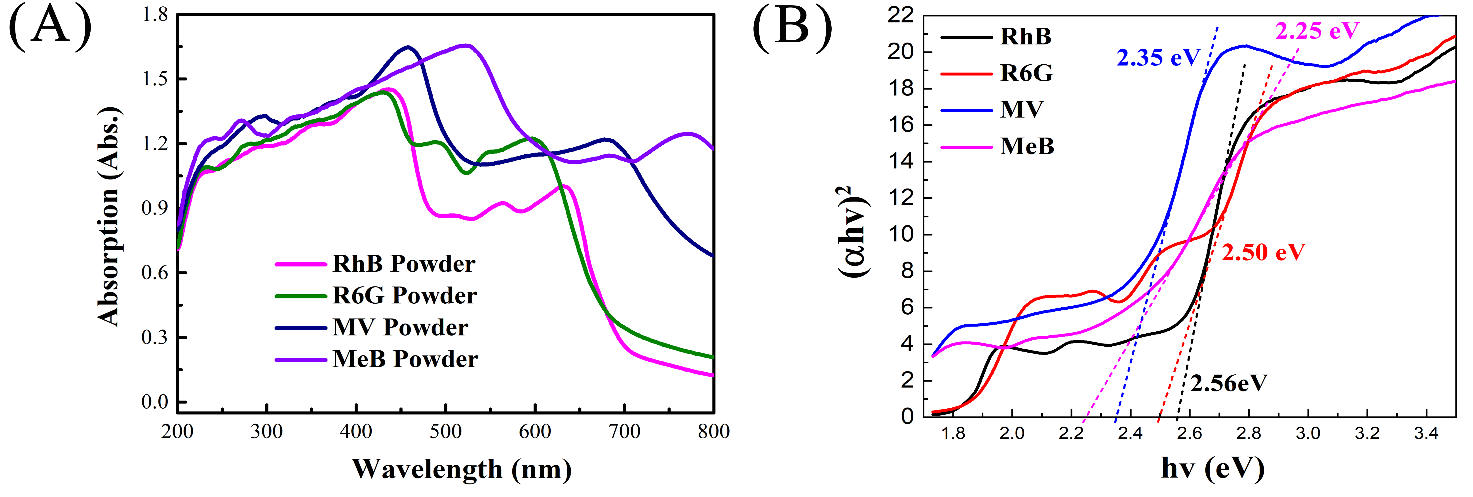


**FIGURE S17. (A)** UV-Vis optical absorption spectra of MeB, MV, R6G, RhB powder. **(B)** Plot of ${(\alpha hv)}^{2}$ as a function of photon energy, Eg, for MeB, MV, R6G, RhB powder.


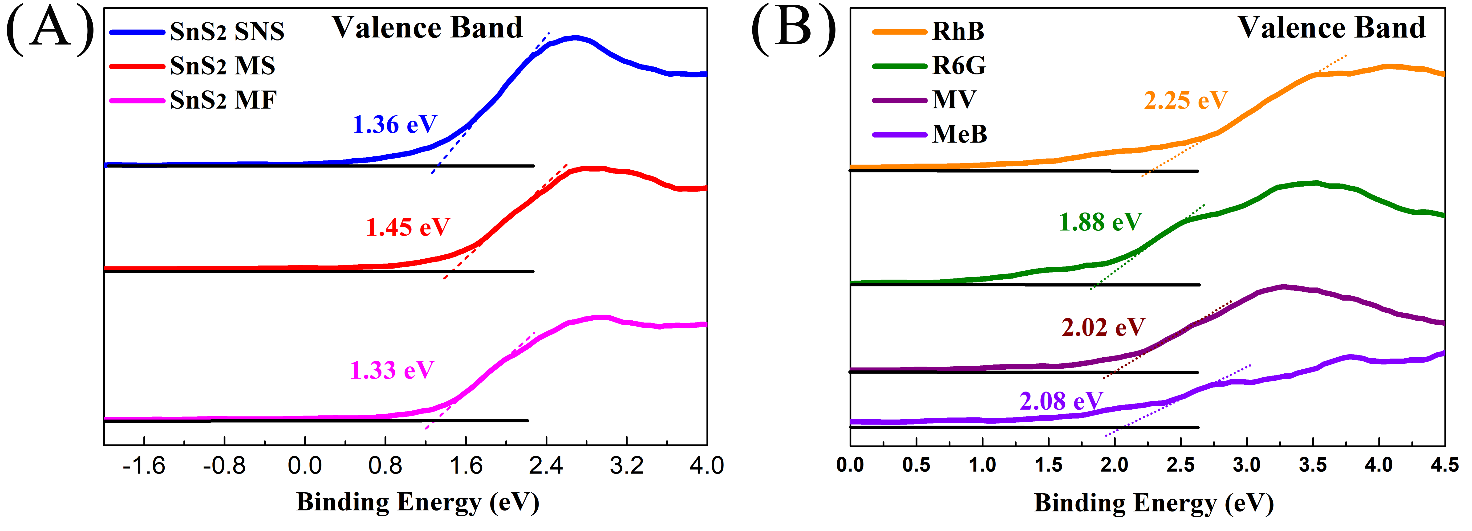


**FIGURE S18. (A-B)** Valence band XPS spectra of for SnS_2_ SNSs, SnS_2_ MSs, SnS_2_ MFs **(A)**, and MeB, MV, R6G, RhB powder **(B)**. The valence band maximum (VBM) are obtained by extrapolating from the emission edge.


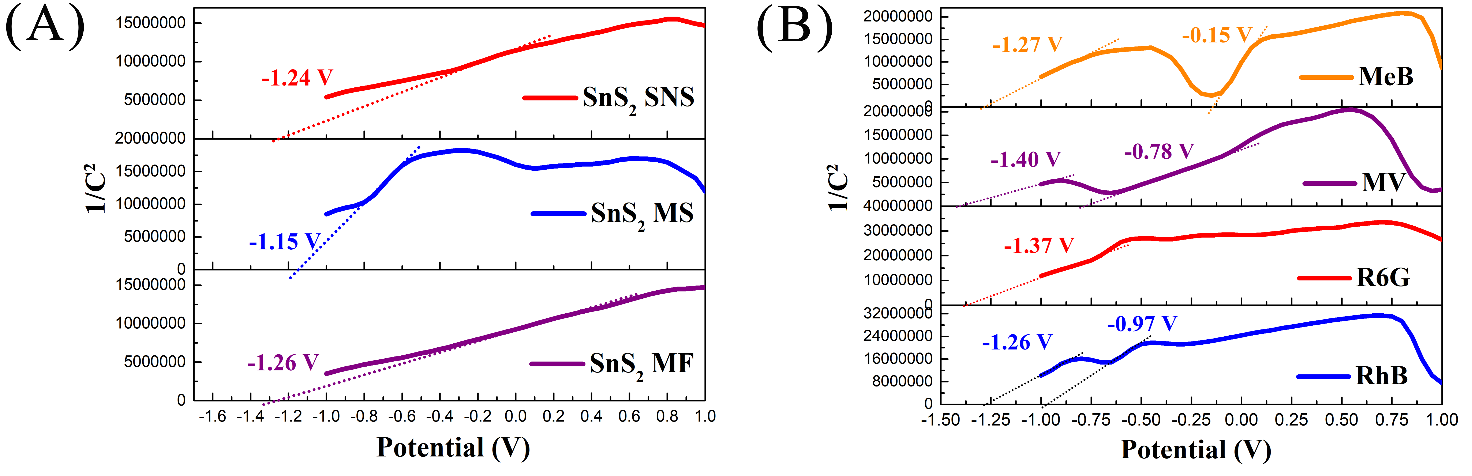


**FIGURE S19. (A-B)** Mott-Schottky plots for SnS_2_ SNSs, SnS_2_ MSs, SnS_2_ MFs **(A)**, and MeB, MV, R6G, RhB powder **(B)**. The flat-band potentials (*V_fb_*) are obtained by extrapolating the curve to the x-axis.

**Table S2.** The measured values of $E_{g}$, $E_{VB}$, $V_{fb}$ and the calculated values of $E_{F, vac}$, $E_{VB, vac}$, $E_{CB, vac}$.

|  | $\boldsymbol{E}_{\boldsymbol{g}}$ | $\boldsymbol{E}_{\boldsymbol{VB}}$ | $\boldsymbol{V}_{\boldsymbol{fb}}$ | $\boldsymbol{E}_{\boldsymbol{F}\boldsymbol{,}\boldsymbol{vac}}$ | $\boldsymbol{E}_{\boldsymbol{VB}\boldsymbol{,}\boldsymbol{vac}}$ | $\boldsymbol{E}_{\boldsymbol{CB}\boldsymbol{,}\boldsymbol{vac}}$ |
| --- | --- | --- | --- | --- | --- | --- |
| **SnS_2_ SNS** | 2.18 eV | 1.36 eV | -1.24 V | -3.465 eV | -4.825 eV | -2.645 eV |
| **SnS_2_ MS** | 1.95 eV | 1.45 eV | -1.15 V | -3.555 eV | -5.005 eV | -3.055 eV |
| **SnS_2_ MF** | 1.90 eV | 1.33 eV | -1.26 V | -3.445 eV | -4.775 eV | -2.875 eV |
| **MeB** | 2.25 eV | 2.08 eV | -1.27 V | -3.435 eV | -5.515 eV | -3.265 eV |
| **R6G** | 2.50 eV | 1.88 eV | -1.37 V | -3.335 eV | -5.215 eV | -2.715 eV |
| **RhB** | 2.56 eV | 2.25 eV | -1.26 V | -3.445 eV | -5.695 eV | -3.135 eV |
| **MV** | 2.35 eV | 2.02 eV | -1.40 V | -3.305 eV | -5.325 eV | -2.975 eV |

**References**

[1] Y. S. Peng, C. L. Lin, Y. Y. Li, Y. Gao, J. Wang, J. He, Z. R. Huang, J. J. Liu, X. Y. Luo, Y. Yang, *Matter* **2022**, *5*, 1.

[2] W. Lee, S. Y. Lee, R. M. Briber, O. Rabin. *Adv. Funct. Mater.* **2011**, *21*, 3424.

[3] V. Dzhagan, N. Mazur, O. Kapush, M. Skoryk, Y. Pirko, A. Yemets, V. Dzhahan, P. Shepeliavyi, M. Valakh, V. Yukhymchuk. *ACS Omega* **2024**, *9*, 4819.

[4] N. V. Mazur, O. A. Kapush, O. F. Isaeva, S. I. Budzulyak, A. Y. Buziashvili, Y. V. Pirko, M. А. Skoryk, A. I. Yemets, O. M. Hreshchuk, V. Yukhymchuk, V. M. Dzhagan, *Physics and Chemistry of Solid State* **2023**, *24*, 682.

[5] A. Virga, P. Rivolo, F. Frascella, A. Angelini, E. Descrovi, F. Geobaldo, F. Giorgis, *J. Phys. Chem. C* **2013**, *117*, 20139.

[6] M. L. Coluccio, G. Das, F. Mecarini, F. Gentile, A. Pujia, L. Bava, R. Tallerico, P. Candeloro, C. Liberale, F. D. Angelis, E. D. Fabrizio, *Microelectron. Eng.* **2009**, *86*, 1085.

[7] H. N. Tran, N. B. Nguyen, N. H. Ly, S. W. Joo, Y. Vasseghian, *Environ. Pollut.* **2023**, *317*, 120775.

[8] I. B. Ansah, S. H. Lee, C. Mun, J. Y. Yang, J. Park, S. Y. Nam, S. Lee, D. H. Kim, S. G. Park, *Sens. Actuators: B. Chem.* **2023**, *379*, 133172.

[9] Y. Yang, Y. Peng, C. Lin, L. Long, J. Hu, J. He, H. Zeng, Z. Huang, Z. Li, M. Tanemura, J. Shi, J. R. Lombardi, X. Luo, *Nano-Micro Lett.* **2021**, *13*, 109.

[10] Y. X. Lin, P. F. Qi, J. C. Liu, Y. Z. Zhuo, C. Cai, H. B. Zhang, X. Y. Li, X. L. Liu, L. Chen, S. H. Luo, X. Y. Tian, *Adv. Mater. Technol.* **2023**, *8*, 2300367.

[11] L. Jiang, X. Y. Wang, J. Y. Zhou, Q. Q. Fu, B. H. Lv, Y. X. Sun, L. P. Song, Y. J. Huang, *Adv. Sci.* **2024**, *11*, 2306125.

[12] W. S. Zhao, S. Yang, D. X. Zhang, T. X. Zhou, J. Huang, M. Gao, X. L. Zhang, Y. Liu, J. H. Yang, *J. Colloid Interf. Sci.* **2023**, *646*, 872.

[13] C. S. H. Hwang, S. Y. Lee, S. J. Lee, H. Kim, T. J. Kang, D. H. Lee, K. H. Jeong, *ACS Appl. Mater. Interfaces* **2022**, *14*, 54550.

[14] J. X. Pei, Z. F. Tian, X. Yu, S. T. Zhang, S. Q. Ma, Y. B. Sun, R. Boukherroub, *Appl. Surf. Sci.* **2023**, *608*, 155270.

[15] C. L. Lin, S. S. Liang, Y. S. Peng, L. Long, Y. Y. Li, Z. R. Huang, N. V. Long, X. Y. Luo, J. J. Liu, Z. Y. Li, Y. Yang, *Nano-Micro Lett.* **2022**, *14*, 75.

[16] S. Z. Qu, Y. X. Zhao, H. S. Kang, J. W. Zou, L. Ma, S. J. Ding, X. B. Chen, *ACS Omega* **2022**, *7*, 48438.

[17] X. Tang, X. Fan, J. Zhou, S. Wang, M. Z. Li, X. Y. Hou, K.W. Jiang, Z. H. Ni, B. Zhao, Q. Hao, T. Qiu, *Nano Lett.* **2023**, *23*, 7037.

[18] Y. S. Peng, C. L. Lin, L. Long, T. Masaki, M. Tang, L. L. Yang, J. J. Liu, Z. R. Huang, Z. Y. Li, X. Y. Luo, J. R. Lombardi, Y. Yang, *Nano-Micro Lett.* **2021**, *13*, 52.

[19] Y. S. Peng, P. Cai, L. L. Yang, Y. Y. Liu, L. F. Zhu, Q. Q. Zhang, J. J. Liu, Z. R. Huang, Y. Yang, *ACS Omega* **2020**, *5*, 26486.

[20] Y. T. Ye, W. C. Yi, W. Liu, Y. Zhou, H. Bai, J. F. Li, G. C. Xi, *Sci. China Mater.* **2020**, *63*, 794.

[21] S. K. Islam, M. Tamargo, R. Moug, J. R. Lombardi, *J. Phys. Chem. C* **2013**, *117*, 23372.

[22] X. T. Wang, W. X. Shi, S. X. Wang, H. W. Zhao, J. Lin, Z. Yang, M. Chen, L. Guo, *J. Am. Chem. Soc.* **2019**, *141*, 5856.

[23] S. Cong, Y. Y. Yuan, Z. G. Chen, J. Y. Hou, M. Yang, Y. L. Su, Y. Y. Zhang, L. Li, Q. W. Li, F. X. Geng, Z. G. Zhao, *Nat. Commun.* **2015**, *6*, 7800.

[24] W. Ji, L. F. Li, W. Song, X. N. Wang, B. Zhao, Y. Ozaki, *Angew. Chem. Int. Ed.* 2019, 58, 14452.

[25] H. Wu, H. Wang, G. Li, *Analyst* **2017**, *142*, 326.

[26] J. Lin, Y. Shang, X. X. Li, J. Yu, X. T. Wang, L. Guo, *Adv. Mater.* **2017**, *29*, 1604797.

[27] L. Yang, Y. S. Peng, Y. Yang, J. J. Liu, H. L. Huang, B. H. Yu, J. M. Zhao, Y. L. Lu, Z. R. Huang, Z. Y. Li, J. R. Lombardi, *Adv. Sci.* **2019**, *6*, 1900310.

[28] Y. S. Peng, C. L. Lin, M. Tang, L. L. Yang, Y. Yang, J. J. Liu, Z. R. Huang, Z. Y. Li, *Appl. Surf. Sci.* **2020**, *509*, 145376.

[29] J. Lin, W. Z. Ren, A. R. Li, C. Y. Yao, T. T. Chen, X. H. Ma, X. T. Wang, A. G. Wu, *ACS Appl. Mater. Interfaces* **2019**, *12*, 4204.

[30] Q. Zhu, S. L. Jiang, K. Ye, W. Hu, J. C. Zhang, X. Y. Niu, Y. X. Lin, S. M. Chen, L. Song, Q. Zhang, J. Jiang, Y. Luo, *Adv. Mater.* **2020**, *32*, 2004059.

[31] X. Song, W. C. Yi, J. F. Li, Q. H. Kong, H. Bai, G. C. Xi, *Nano Lett.* **2021**, *21*, 4410.

[32] H. M. Guan, W. C. Yi, T. Li, Y. H. Li, J. F. Li, H. Bai, G. C. Xi, *Nat. Commun.* **2020**, *11*, 3889.

[33] K. K. Wang, Z. Y. Guo, Y. Li, Y. X. Guo, H. Liu, W. Zhang, Z. Z. Zou, Y. L. Zhang, Z. M. Liu, *ACS Appl. Nano Mater.* **2020**, *3*, 11363.

[34] Y. N. Quan, J. C. Yao, Y. S. Sun, X. Qu, R. Su, M. Y. Hu, L. Chen, Y. Liu, M. Gao, J. H. Yang, *Sens. Actuators B Chem.* **2021**, *327*, 128903.
